# Functional connectome differences in individuals with hallucinations across the psychosis continuum

**DOI:** 10.1038/s41598-020-80657-8

**Published:** 2021-01-13

**Authors:** Maya J. L. Schutte, Marc M. Bohlken, Guusje Collin, Lucija Abramovic, Marco P. M. Boks, Wiepke Cahn, Meenakshi Dauwan, Edwin van Dellen, Neeltje E. M. van Haren, Kenneth Hugdahl, Sanne Koops, René C. W. Mandl, Iris E. C. Sommer

**Affiliations:** 1 Department of Biomedical Sciences of Cells and Systems, University of Groningen, University Medical Center Groningen, Neuroimaging Center, PO Box 196, 9700 AD Groningen, The Netherlands; 2 Department of Psychiatry, University Medical Center Utrecht Brain Center, Utrecht University, Utrecht, The Netherlands; 3 Department of Psychiatry, Beth Israel Deaconess Medical Center and Massachusetts Mental Health Center, Harvard Medical School, Boston, MA USA; 4 McGovern Institute for Brain Research, Department of Brain and Cognitive Sciences, Massachusetts Institute of Technology, Cambridge, MA USA; 5 Department of Intensive Care Medicine and UMC Utrecht Brain Center, University Medical Center Utrecht, Utrecht University, Utrecht, The Netherlands; 6 Department of Child and Adolescent Psychiatry, Erasmus Medical Centre, Rotterdam, The Netherlands; 7 Department of Biological and Medical Psychology, University of Bergen, Bergen, Norway; 8 Department of Psychiatry, Haukeland University Hospital, Bergen, Norway; 9 Department of Radiology, Haukeland University Hospital, Bergen, Norway; 10 NORMENT Center for the Study of Mental Disorders, University of Oslo, Oslo, Norway

**Keywords:** Neuroscience, Psychology

## Abstract

Hallucinations may arise from an imbalance between sensory and higher cognitive brain regions, reflected by alterations in functional connectivity. It is unknown whether hallucinations across the psychosis continuum exhibit similar alterations in functional connectivity, suggesting a common neural mechanism, or whether different mechanisms link to hallucinations across phenotypes. We acquired resting-state functional MRI scans of 483 participants, including 40 non-clinical individuals with hallucinations, 99 schizophrenia patients with hallucinations, 74 bipolar-I disorder patients with hallucinations, 42 bipolar-I disorder patients without hallucinations, and 228 healthy controls. The weighted connectivity matrices were compared using network-based statistics. Non-clinical individuals with hallucinations and schizophrenia patients with hallucinations exhibited increased connectivity, mainly among fronto-temporal and fronto-insula/cingulate areas compared to controls (*P* < 0.001 adjusted). Differential effects were observed for bipolar-I disorder patients with hallucinations versus controls, mainly characterized by decreased connectivity between fronto-temporal and fronto-striatal areas (*P* = 0.012 adjusted). No connectivity alterations were found between bipolar-I disorder patients without hallucinations and controls. Our results support the notion that hallucinations in non-clinical individuals and schizophrenia patients are related to altered interactions between sensory and higher-order cognitive brain regions. However, a different dysconnectivity pattern was observed for bipolar-I disorder patients with hallucinations, which implies a different neural mechanism across the psychosis continuum.

## Introduction

Hallucinations are a hallmark feature of schizophrenia, but occur in a range of psychiatric, neurological and general medical conditions^1^, and even in a minority of healthy individuals^2^. Hallucinations, and other psychotic phenomena, can be understood to exist along a continuum, ranging from subclinical symptoms in healthy individuals on one end, to individuals with a severe psychotic illness on the other^3^. Patients with bipolar disorder who experience psychotic symptoms may hold a position in between both ends of the spectrum. However, it remains to be determined if hallucinations in non-clinical individuals, patients with schizophrenia, and patients with bipolar disorder share a common neural mechanism (and differ mainly in severity) or if hallucinations stem from different neural mechanisms across these three groups.

Functional magnetic resonance imaging (fMRI) has proven to be a powerful tool to investigate neural mechanisms associated with hallucinations. Studies have shown elevated levels of activity in sensory cortices during hallucinations in schizophrenia^4,5^, suggesting inadequate higher-order control over activity in these areas. Cognitive control is mediated by cognitive regions, including areas of the default-mode, central executive, and task-positive network^6–9^. Alterations in these networks have therefore been linked to hallucinations^7^. Furthermore, the insula and (para)hippocampal areas have been related to hallucinations^9–12^ suggesting the involvement of salience and memory processes. Taken together, hallucinations may arise from abnormal connectivity patterns among several areas in the functional connectome^13–15^.

The question arises if these findings in schizophrenia can be extended to hallucinations in other phenotypic groups. Hallucinations in healthy individuals and patients with bipolar disorder show partly overlapping phenomenological features with hallucinations in schizophrenia^16–18^, although differences in frequency, duration, and emotional content are consistently described^16,19^. In addition, neuropsychological studies indicate that cognitive control processes, including inhibition and executive functioning, are less altered in bipolar-I patients than in schizophrenia patients and may be used for compensatory strategies^20–22^.

This study sets out to compare functional connectome alterations between individuals with hallucinations across the psychosis continuum. To this end, we included patients with a schizophrenia spectrum disorder, bipolar-I disorder with and without a lifetime history of hallucinations, and healthy controls with and without hallucinations. Using a network-based approach, we examined whole-brain functional interactions between brain regions, allowing us to study whether similar neural mechanisms are present across diagnostic groups.

Based on the notion that hallucinations are related to dysconnectivity, we hypothesize that the functional connectome of *all* individuals with hallucinations will show widespread alterations, particularly in sensory areas including the auditory and visual cortex, limbic areas related to salience processing, and frontal areas related to cognitive control.

## Materials and methods

### Participants

This cross-sectional study included 116 patients with bipolar-I disorder, including 74 with a lifetime history of hallucinations (BD-H) and 42 without a lifetime history of psychotic experiences (BD), 99 patients with a schizophrenia spectrum disorder, all of whom endorsed lifetime hallucinations (SCZ-H), 40 non-clinical individuals with hallucinations (NC-H), and 228 healthy controls without any history of hallucinations (HC). Participants were recruited as part of five different studies that were all conducted at the University Medical Center Utrecht. See Supplementary Methods for more information on recruitment and characterization. The studies were approved by the Institutional Review Board of the University Medical Center Utrecht. All participants provided written informed consent. All methods and study procedures were conducted in accordance with the relevant guidelines and regulations. Presence of current or lifetime hallucinations and assessment of hallucination modality was established using the Comprehensive Assessment of Symptoms And History (CASH^23^). Hallucination severity was determined with the Positive and Negative Syndrome Scale (PANSS^24^) in patients with schizophrenia, and with the Psychotic Symptoms Rating Scales (PSYRATS^25^) in non-clinical individuals. Hallucination severity was not assessed in patients with bipolar-I disorder, as none of these participants experienced current hallucinations (see Table 1).

**Table 1:** Participant characteristics on demographic and clinical variables (n = 483).

	SCZ-H (*n* = 99)	BD-H (*n* = 74)	BD (*n* = 42)	NC-H (*n* = 40)	HC (*n* = 228)	*P* value
Age, mean (SD), years	32.3 (10.9)	46.2 (11.6)	52.3 (11.9)	43.1 (14.5)	40.7 (14.6)	< 0.001*
Gender, m/f	67/32	37/37	23/19	10/30	121/107	< 0.001*
Handedness, R/L	85/8	65/8	36/7	30/10	190/37	0.106
**Motion, mm**						
Relative mean displacement	0.1 (0.04)	0.1 (0.03)	0.1 (0.03)	0.08 (0.03)	0.08 (0.03)	< 0.001*
**Diagnosis, No. (%)**						
Schizophrenia	59 (59.6)					
Schizoaffective disorder	17 (17.2)					
Schizophreniform disorder	1 (1.0)					
Schizotypal personality	1 (1.0)					
Psychosis NOS	20 (20.2)					
Bipolar-I disorder		74 (100)	74 (100)			
**Medication, No. (%)**						
Antipsychotics	83 (83.8)	39 (52.7)	9 (21.4)	0 (0.0)	0 (0.0)	
Lithium	5 (5.1)	42 (56.8)	27 (64.3)	0 (0.0)	0 (0.0)	
Anti-depressants	22 (22.2)	16 (21.6)	10 (23.8)	2 (5.0)	3 (1.3)	
**Hallucinations lifetime, No. (%)**	99 (100)	74 (100)	0 (0.0)	40 (100)	0 (0.0)	
Auditory	80 (80.8)	39 (52.7)	0 (0.0)	40 (100)	0 (0.0)	< 0.001*
Visual	53 (53.5)	51 (68.9)	0 (0.0)	33 (82.5)	0 (0.0)	< 0.001*
**Hallucinations current, No. (%)**	57 (65.5)	0 (0.0)	0 (0.0)	40 (100)	0 (0.0)	
**Delusions lifetime, No. (%)**	72 (72.7)	64 (86.5)	0 (0.0)	14 (35.0)	1 (0.4)	

Differences between continuous variables were tested with a one-way ANOVA, and differences in dichotomous variables with Chi-square tests. Significance was set at P < 0.05. Significant differences are indicated by an asterisk (*). Current hallucinations means experiences in the last month. The current hallucinations in the SCZ-H were based on the P3 > 2 PANSS, and in NC-H with the frequency item of the PSYRATS. Number of cases with missing data: Diagnosis n = 1 SCZ-H; Antipsychotics n = 10 HC; n = 5 SCZ-H; n = 3 BD-H; n = 1 BD. Lithium n = 10 HC; n = 6 SCZ-H. Antidepressants; n = 10 HC; n = 16 SCZ-H; Lifetime hallucinations n = 4 HC; Auditory n = 4 HC; n = 11 SCZ-H; n = 2 BD-H. Visual n = 5 HC; n = 15 SCZ-H. Olfactory n = 5 HC; n = 17 SCZ-H. Tactile n = 5 HC; n = 14 SCZ-H. Current hallucinations: SCZ-H = 23. Lifetime delusions; n = 5 HC; n = 3 NC-H; n = 21 SCZ-H.

*NC-H* non-clinical individuals with hallucinations, *NOS* not otherwise specified, *BD-H* bipolar-I disorder with lifetime history of hallucinations, *BD* bipolar-I disorder without lifetime history of hallucinations, *SCZ-H* schizophrenia spectrum disorder with hallucinations.

### Data acquisition and preprocessing

See Supplementary Methods for a detailed description of acquisition parameters, preprocessing steps and motion correction.

### Functional network construction

Average timeseries for each participant were extracted from N = 90 functional regions (nodes) of the automated anatomical labeling (AAL) atlas^26^ and N = 260 nodes of the Power atlas^27^ covering cortical and subcortical brain regions, but not the cerebellum. Wavelet decompositions to each of the regional time series were applied, extracting wavelet coefficients in scale 4 (0.05–0.10 Hz)^28,29^. Wavelet filtering was done by the maximal overlap discrete wavelet transform (MODWT) method using the WMSTA toolbox in Matlab (http://www.atmos.washington.edu/~wmtsa/). Functional connectivity was estimated between wavelet coefficients of any pair of regions *i* and *j* (edges) using wavelet coherence, done by the *mscohere* function in Matlab. See the Supplementary Material for more details on wavelet coherence.

### Global connectome alterations

We analysed alterations in global network organization related to hallucinations. A detailed description of the graph measures can be found in the Supplemental Methods.

### Network based statistics

A Network-Based Statistic (NBS) analysis^30,31^ was used to obtain information about the localization of connectivity alterations in the overall connectome. Interconnected components of altered connections were identified within the overall connectome using two-sided F-tests to explore increased and decreased levels of functional connectivity (instead of multiple one-sided *t*-tests). A Family Wise Error (FWE) adjusted *P*-value was calculated for each component using permutation testing (10,000 permutations) to determine the likelihood that a component of this size could arise by chance^30^. We compared all four participant groups (NC-H, SCZ-H, BD-H, BD) to controls (HC) in order to investigate if similar alterations are present in the hallucination groups, and if different alterations would be observed for the bipolar-I patients *without* hallucinations. We conducted these analyses for both the structural AAL-90 and functional Power-260 atlas, as there is some controversy regarding the use of a structural atlas in functional connectivity analyses.

Following Fornito et al.^32^, we organized all nodes of the AAL atlas into their corresponding lobes, and calculated the proportion of altered connections between each pair of lobes to pinpoint the lobes with the most alterations. In order to obtain more specific regional information on the most important nodes in the network, we ranked the nodes in the NBS networks on node degree per group, similar to Zalesky et al.^34^. In addition, a conjunction analysis was conducted to test for overlap of connections in each NBS network.

### K-means clustering

To directly test for differences in dysconnectivity between the hallucination groups, we compared the three hallucination groups (NC-H, SCZ-H, and BD-H) in one NBS contrast. To help interpret the findings of this analysis, the connections in the resulting component were fed into a k-means clustering analysis (implemented in Matlab)^33^. This allowed us to cluster connections that behaved either similarly or different in terms of decreased or increased connectivity across the three hallucination groups (i.e. NC-H, SCZ-H, BD-H) (see Supplemental Material).

### Symptom correlations

To test for associations with hallucination severity, PANSS item P3-Hallucinations was assessed for correlations with global network metrics using a non-parametric Spearman correlation within the schizophrenia group. Similarly, PSYRATS items frequency and severity of auditory hallucinations were explored for associations with network metrics in the non-clinical group. To correct for multiple testing, correlations are reported at *P* < 0.05 false discovery rate (FDR).

### Hallucination modality

A significant difference was found in the reported hallucination modality across the three hallucination groups (χ^2^(4) = 366.0, P < 0.001 for auditory; χ^2^(4) = 265.3, P < 0.001 for visual, see Table 1). To explore effects of hallucination modality, we replicated the NBS analyses in a subgroup of participants only experiencing auditory hallucinations. We compared a subgroup of bipolar-I disorder patients who all only experienced auditory hallucinations (n = 39), to healthy controls (n = 228), as well as schizophrenia patients with only auditory hallucinations (n = 28) versus healthy controls (n = 228). Repeating our analyses in the non-clinical group with *only* auditory hallucinations was not feasible, as n = 7 participants experienced only auditory hallucinations.

Furthermore, we applied a random forest classification algorithm (part of the BrainWave software https://home.kpn.nl/stam7883/brainwave.html) to identify a set of connections in each NBS network that could differentiate between the experience of having either auditory or visual hallucinations within each group (i.e. NC-H, SCZ-H and BD-H). Each decision tree in the random forest is built using a bootstrap sample (thus with replacement), from the original data. Both bootstrap aggregating (i.e., bagging) and random feature selection avoid overfitting and reduce variance in the model, which results in uncorrelated trees^34^. Hence, the random forest classifier has the advantage that cross-validation is done internally, meaning that no separate test set of participants is needed to estimate the generalized error of the training set^34^.

The random forest parameters, mTry (i.e., the number of input variables chosen randomly at each split calculated by the square root of number of features) and nTree (i.e., the number of trees to grow for each forest) were set to 9 and 500, respectively.

In every classification, each feature is given a variable importance (VIMP) score between 0 and 1. Weighted accuracy, sensitivity and specificity were used to assess the random forest for its capability to distinguish between visual and auditory hallucinations. We computed weighted accuracies to correct for unequal group sizes^35^, see Supplementary Methods for more details on the random forest classifier.

Features fed into the classifier were selected based on their occurrence in the NBS network per group. The edges as presented in Supplementary Tables 13–15 were included for each of the groups. The number in front of each edge pair corresponds to the number of each edge pair in Supplementary Fig. 7. Two random forest parameters that need to be entered into the model were set at mTry = 9; and nTree = 500. mTry is the square root number of features, and the nTree is the number of trees to grow for each forest.

Note that in schizophrenia patients and non-clinical individuals, the condition ‘visual hallucinations’ means that participants experienced both auditory and visual hallucinations (the largest proportion of participants all endorsed auditory hallucinations, see Table 1). The condition ‘auditory hallucinations’ means that schizophrenia patients and non-clinical individuals experienced only auditory hallucinations. A clearer distinction between *only* auditory and *only* visual could be made in bipolar-I disorder patients. Please see Supplementary Methods for more information on cross validation of the random forest classifier.

### Confounding effects

Due to differences in demographic variables and motion parameters, several validation analyses were conducted and reported in the Supplementary Methods and Results. A quality check of distance dependent effects of motion on edge strength per participant group can be found in Supplementary Fig. 1.

## Results

### Participant characteristics

A total of 483 participants were included in the analyses; see Table 1 for demographic and clinical characteristics, and Supplementary Tables 1, 2 for more information on exclusion of subjects (e.g. due to effects of motion). Schizophrenia patients were primarily characterized by lifetime auditory (80.8%) and visual (53.5%) hallucinations. Non-clinical individuals with hallucinations all experienced lifetime auditory hallucinations (100%) and the large majority (82.5%) also experienced visual hallucinations. Conversely, bipolar-I disorder patients mostly experienced visual hallucinations (68.9%), whereas about half (52.7%) experienced auditory hallucinations.

In line with previous studies, there was a higher proportion of males in the schizophrenia group, and a higher proportion of females in the non-clinical group (χ^2^(4) = 21.5, *P* < 0.001). A significant difference for age was found (F(4) = 21.7, *P* < 0.001), with the schizophrenia group being younger and bipolar-I disorder group being older than other three groups (post-hoc tests in Supplementary Tables 3, 4). As age and sex were not collinear, these variables were added as covariates in all analyses (see Supplementary Results). The groups also differed in motion parameters (F(4) = 20.3, *P* < 0.001), with patients with bipolar-I disorder and schizophrenia showing more movement than healthy controls and non-clinical individuals. Additional analyses revealed a minimal influence of motion on connectivity measures (see Supplementary Fig. 1).

### Global connectome alterations

There were no significant differences in metrics of global network topology between the groups, see the Supplemental Results for more information.

### Network based statistics

**Figure 1:**
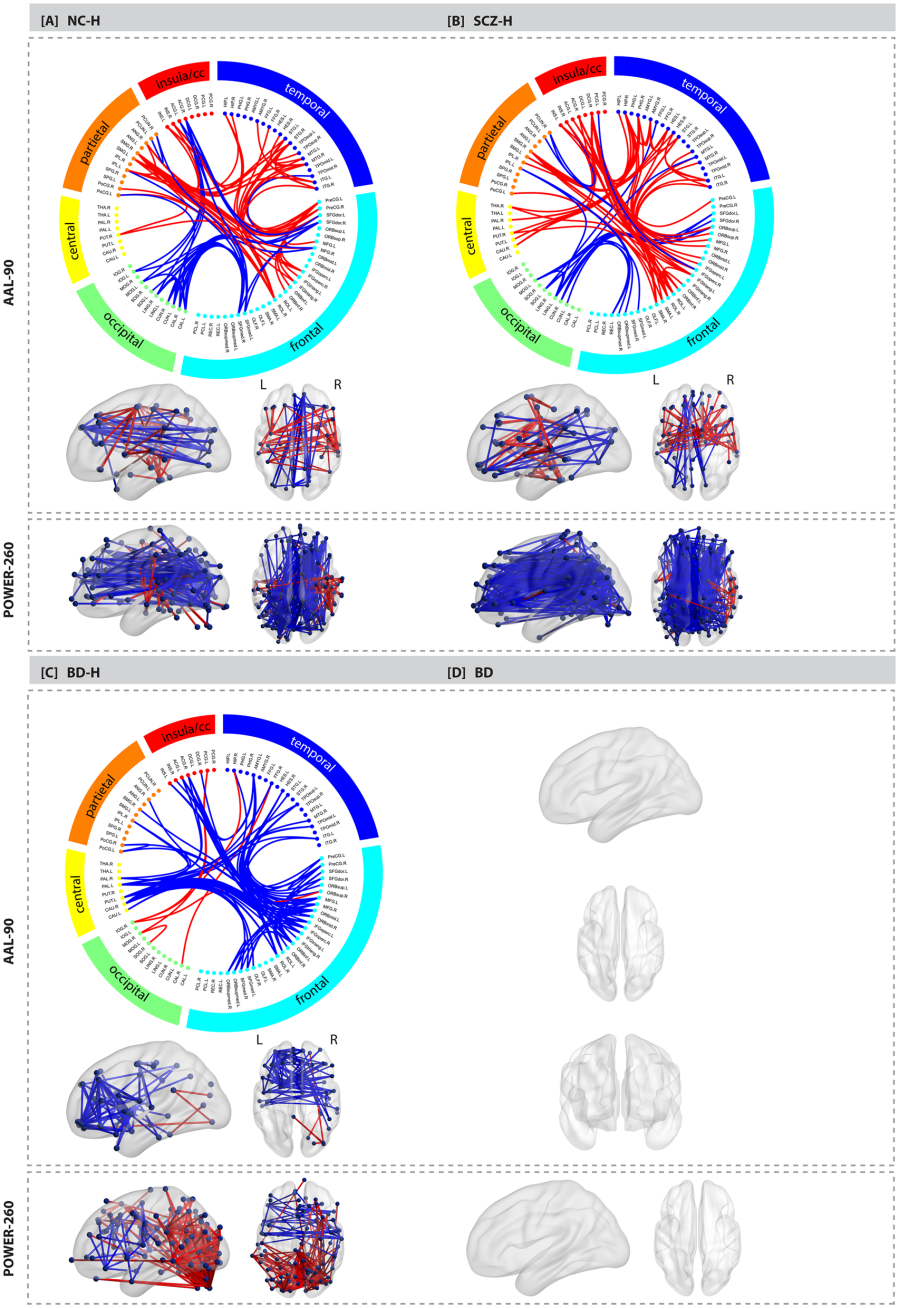
Functional connectome alterations in the non-clinical and clinical groups compared to healthy controls. The Network Based Statistics results of the AAL atlas are depicted in de upper grey panel and the results of the Power atlas in the lower grey panel: (**a**) non-clinical individuals with hallucinations (n = 40) vs healthy controls (n = 228); (**b**) schizophrenia patients with hallucinations (n = 99) vs healthy controls (n = 228); (**c**) bipolar-I disorder patients with lifetime history of hallucinations (n = 74) vs healthy controls (n = 228). (**d**) bipolar-I disorder patients without a lifetime history of psychosis (n = 42) did not differ from controls (n = 228). The edges are color-coded based on either an increase (red) or decrease (blue) in connectivity compared to controls. The circle plot depicts all 90 nodes of the AAL atlas clustered according to their cerebral lobe. Group differences were tested at 10,000 permutations *P* < 0.05 FWE corrected. Age and sex were included as covariates. The corresponding test-statistics, a list of altered connections (Supplementary Tables 6–8 for AAL atlas, Supplementary Tables 10–12 for Power atlas), and abbreviations for the AAL brain regions depicted in the circle plot (Supplementary Table 16) can be found in the Supplementary. *BD* bipolar-I disorder without lifetime history of hallucinations, *BD-H* bipolar-I disorder with lifetime history of hallucinations, *CC* cingulate cortex, *NC-H* non-clinical individuals with hallucinations, *SCZ-H* schizophrenia spectrum disorder with hallucinations.

When separately comparing each hallucination group to controls, schizophrenia patients and non-clinical individuals with hallucinations exhibited a similar pattern of increased and decreased connectivity between a wide range of brain areas (*P* < 0.001 FWE corrected, for both; see Fig. 1A,B), whereas bipolar-I disorder patients with hallucinations revealed a markedly different pattern of mostly decreased connectivity (*P* = 0.012 FWE corrected; see Fig. 1C). Bipolar-I disorder patients without hallucinations did not exhibit significant alterations in connectivity relative to controls (*P* > 0.05, FWE corrected; see Fig. 1D). For a complete list of connections in each NBS network, see Supplementary Tables 6–8 for the AAL atlas and Supplementary Tables 10–12 for the Power atlas. These results were broadly replicated in matched subgroups for the AAL atlas, when age and sex were not included as covariates, see Supplementary Fig. 2.

**Figure 2:**
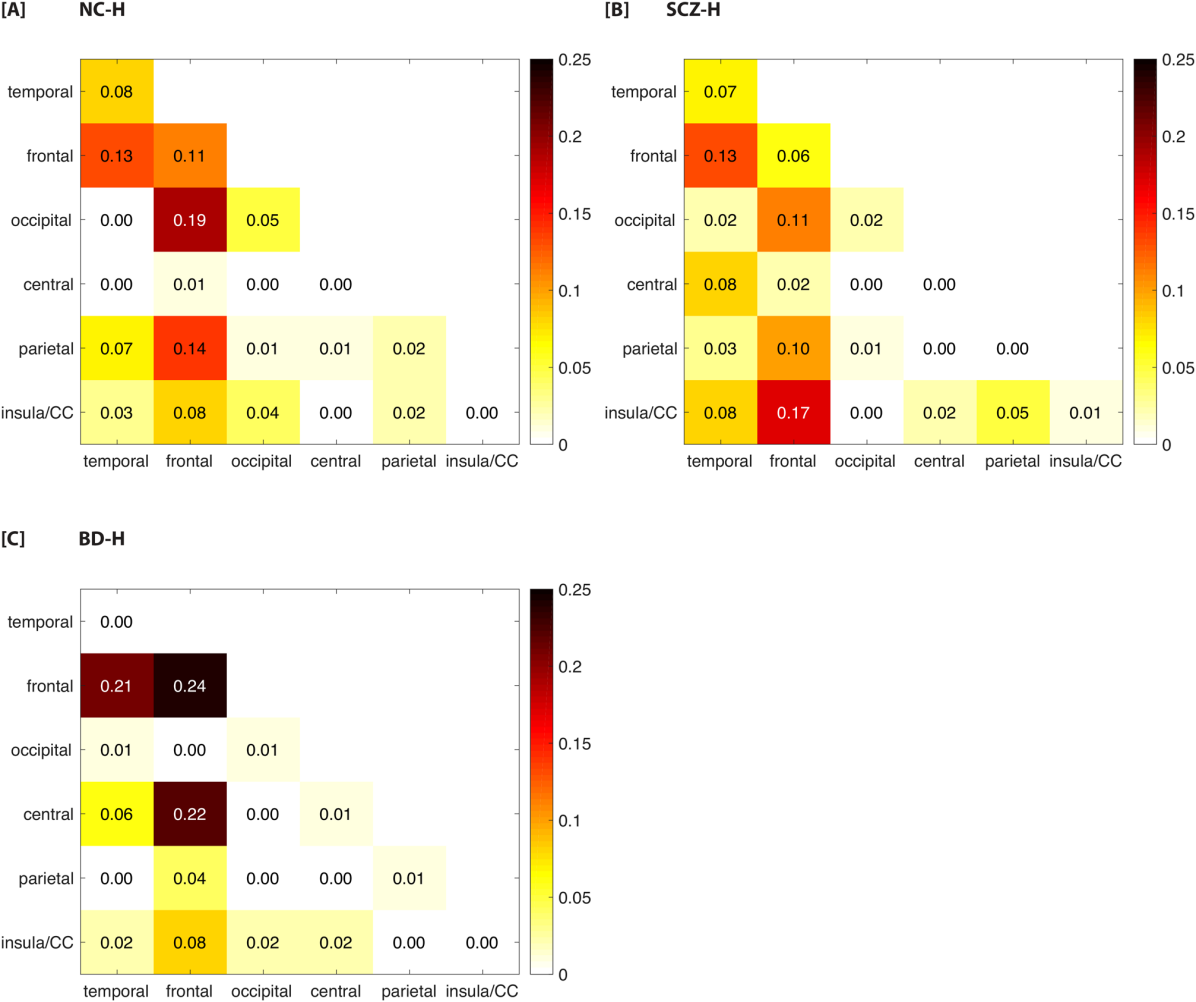
Proportion of altered connections between each pair of lobes for the three hallucination groups relative to controls. The number of altered links between each pair of lobes was divided by the total number of altered pair-wise links based on the results of the AAL atlas. (**a**) non-clinical individuals with hallucinations (n = 40) vs healthy controls (n = 228); (**b**) schizophrenia patients with hallucinations (n = 99) vs healthy controls (n = 228); (**c**) bipolar-I disorder patients with lifetime history of hallucinations (n = 74) vs healthy controls (n = 228). Note that the hippocampus and amygdala are assigned to the temporal lobe. The putamen, pallidum and caudate are included in the central lobe. *BD* bipolar-I disorder without lifetime history of hallucinations, *BD-H* bipolar-I disorder with lifetime history of hallucinations, *CC* cingulate cortex, *NC-H* non-clinical individuals with hallucinations, *SCZ-H* schizophrenia spectrum disorder with hallucinations.

Using the AAL atlas, the majority of connections comprising the NBS components in schizophrenia patients and non-clinical individuals were found between fronto-insula/cingulate regions, followed by fronto-temporal, temporo-temporal, fronto-occipital, and fronto-parietal alterations (see Fig. 2A,B). The NBS subnetwork in schizophrenia patients included more altered connections among fronto-insula/cingulate and temporal-central regions whereas altered fronto-occipital connections were more prevalent in non-clinical individuals. Similar alterations were found when using the Power atlas, including increased connectivity between the auditory, sensorimotor and cingulo-opercular control network (see Fig. 1 and Supplementary Fig. 3a,b for a circle plot of the results for the Power atlas). Decreased connectivity was found mainly between the visual, default mode, central executive and ventral attention networks in both groups.

For bipolar-I disorder patients with hallucinations, the most pronounced alterations were found among fronto-temporal, fronto-central connections and between the bilateral frontal lobes when using the AAL atlas (see Fig. 2). These results were replicated using the Power atlas, reporting increased connectivity between the visual, default mode, subcortical and dorsal attention networks (see Fig. 1C and Supplementary Fig. 3C). Decreased connectivity was found between the auditory, sensorimotor, cingulo-opercular, salience and central-executive network.

In schizophrenia patients, the node with the highest number of altered connections in the NBS component was the left middle cingulate gyrus (see Table 2), whereas this was the right superior medial frontal gyrus for non-clinical individuals, and left amygdala for bipolar-I disorder patients with hallucinations.

**Table 2:** Node degree of NBS network per group.

NC-H (n = 40)		SCZ-H (n = 99)		BD-H (n = 74)	
Node	Degree	Node	Degree	Node	Degree
Frontal_Sup_Medial_R	12	Cingulum_Mid_R	17	Frontal_Mid_Orb_L	19
Precentral_L	12	Hippocampus_R	10	Amygdala_L	13
Rolandic_Oper_R	11	Frontal_Sup_Orb_L	9	Caudate_R	12
SupraMarginal_R	11	Rolandic_Oper_R	9	Frontal_Mid_L	12
Temporal_Sup_R	10	Rolandic_Oper_L	8	Frontal_Inf_Orb_L	10
Frontal_Sup_R	8	SupraMarginal_R	6	Pallidum_L	9
Cingulum_Ant_R	7	Frontal_Inf_Oper_R	5	Putamen_L	9
Lingual_R	7	Heschl_R	5	Frontal_Inf_Oper_R	6
Calcarine_L	6	Lingual_L	5	Precentral_R	6
Cuneus_L	6	Occipital_Mid_L	5	Rolandic_Oper_R	6
Frontal_Mid_R	5	ParaHippocampal_R	5	Rolandic_Oper_L	5
Frontal_Sup_Medial_L	5	Temporal_Sup_L	5	Cingulum_Ant_L	4
Temporal_Mid_R	5	Amygdala_R	4	Cingulum_Ant_R	4
Cuneus_R	4	Cingulum_Ant_R	4	Frontal_Inf_Oper_L	4
Frontal_Sup_L	4	Frontal_Inf_Tri_L	4	Frontal_Mid_R	4
Insula_R	4	Cuneus_R	3	Temporal_Pole_Sup_L	4
Parietal_Inf_L	4	Heschl_L	3	Frontal_Inf_Tri_L	3
Precentral_R	4	Insula_L	3	Frontal_Med_Orb_R	3
Temporal_Sup_L	4	Insula_R	3	Frontal_Sup_Medial_L	3
Angular_R	3	Occipital_Sup_L	3	Heschl_R	3
Calcarine_R	3	Parietal_Inf_L	3	Occipital_Inf_R	3
Frontal_Inf_Oper_R	3	Supp_Motor_Area_R	3	Parietal_Inf_R	3
Insula_L	3	Temporal_Inf_L	3	Caudate_L	2
Occipital_Mid_L	3	Caudate_R	2	Cingulum_Post_L	2
SupraMarginal_L	3	Cingulum_Ant_L	2	Frontal_Med_Orb_L	2
Temporal_Inf_L	3	Frontal_Inf_Orb_R	2	Frontal_Mid_Orb_R	2
Temporal_Inf_R	3	Frontal_Sup_L	2	Frontal_Sup_L	2
Cingulum_Ant_L	2	Pallidum_R	2	Frontal_Sup_Medial_R	2
Frontal_Inf_Tri_R	2	Postcentral_L	2	Frontal_Sup_Orb_R	2
Hippocampus_L	2	Precuneus_R	2	Frontal_Sup_R	2
Occipital_Inf_L	2	Putamen_L	2	Fusiform_R	2
Postcentral_L	2	Putamen_R	2	Hippocampus_R	2
Precuneus_R	2	Rectus_L	2	Insula_L	2
Putamen_R	2	Supp_Motor_Area_L	2	Occipital_Mid_R	2
Temporal_Mid_L	2	SupraMarginal_L	2	Olfactory_R	2
Amygdala_R	1	Temporal_Inf_R	2	Pallidum_R	2
Frontal_Inf_Orb_L	1	Temporal_Mid_L	2	Postcentral_L	2
Frontal_Inf_Orb_R	1	Thalamus_R	2	Postcentral_R	2
Frontal_Inf_Tri_L	1	Cingulum_Post_L	1	Precentral_L	2
Frontal_Mid_L	1	Cuneus_L	1	Temporal_Inf_L	2
Fusiform_R	1	Frontal_Inf_Oper_L	1	Temporal_Pole_Mid_L	2
Heschl_R	1	Frontal_Inf_Orb_L	1	Angular_R	1
Hippocampus_R	1	Frontal_Med_Orb_L	1	Calcarine_R	1
Occipital_Sup_L	1	Frontal_Med_Orb_R	1	Cingulum_Mid_R	1
Postcentral_R	1	Frontal_Mid_L	1	Frontal_Inf_Orb_R	1
Precuneus_L	1	Frontal_Mid_Orb_R	1	Frontal_Inf_Tri_R	1
Temporal_Pole_Mid_R	1	Frontal_Mid_R	1	Frontal_Sup_Orb_L	1
Temporal_Pole_Sup_R	1	Frontal_Sup_Medial_L	1	Insula_R	1
		Frontal_Sup_Orb_R	1	Occipital_Sup_R	1
		Frontal_Sup_R	1	SupraMarginal_R	1
		Fusiform_L	1	Temporal_Mid_R	1
		Hippocampus_L	1	Temporal_Sup_L	1
		Parietal_Inf_R	1		
		Precentral_L	1		
		Precuneus_L	1		
		Temporal_Mid_R	1		
		Temporal_Pole_Sup_R	1		

*NC-H* non-clinical individuals with hallucinations, *BD-H* bipolar-I disorder with lifetime history of hallucinations, *SCZ-H* schizophrenia spectrum disorder with hallucinations.

The conjunction analysis revealed considerable variation across the exact connections comprising the respective NBS networks, see Supplementary Fig. 4 and Supplementary Table 5 for a list of overlapping connections.

### K-means clustering

**Figure 3:**
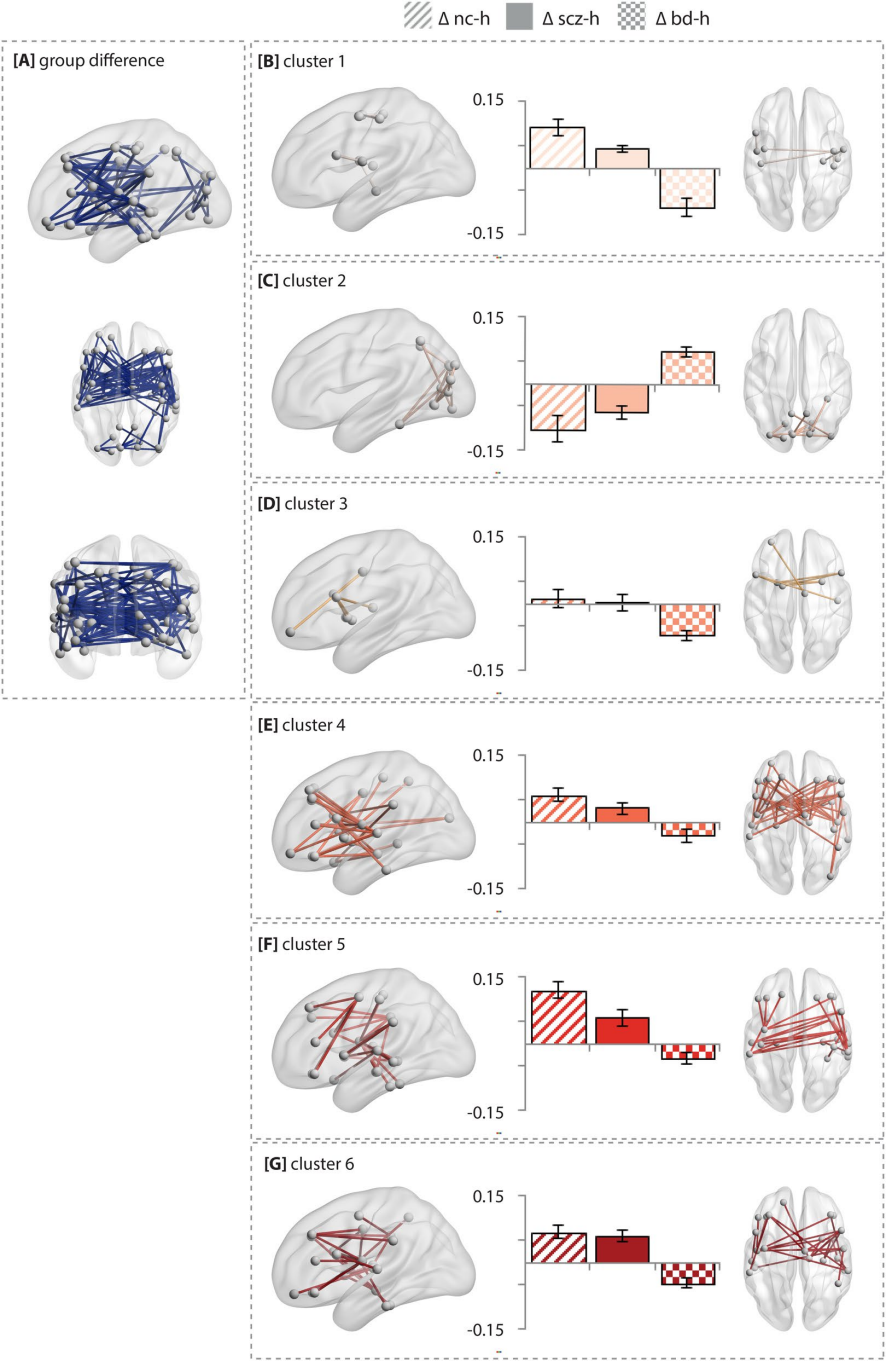
Sets of connections clustered according to their behavior in clinical and non-clinical individuals with hallucinations. (**A**) The result of the overall group comparison between patients with schizophrenia (n = 99), patients with bipolar-I disorder with (n = 74) and non-clinical individuals with hallucinations (n = 40) using the AAL atlas. (**B–E**) the edges in the component that differed between the groups were clustered by a k-means clustering algorithm to elucidate differences across the groups with hallucinations. Error bars represent the standard deviation of connectivity strength within that particular cluster. The group differences were tested using an F-test at T = 8.0; *P* < 0.05 at 10,000 permutations. The test-statistics and AAL labels corresponding to the altered connections per cluster can be found in Supplementary Table 9. Age and sex were included as covariates. *NC-H* non-clinical individuals with hallucinations, *BD-H* bipolar-I disorder with lifetime history of hallucinations, *SCZ-H* schizophrenia spectrum disorder with hallucinations.

A direct NBS comparison of the three hallucination groups yielded a subnetwork of connections that differed between non-clinical individuals, schizophrenia patients, and bipolar-I patients with hallucinations (*P* < 0.001; see Fig. 3A and Supplementary Table 9 for a list of connections). To help interpret the resulting subnetwork, a k-means clustering analysis was performed, which yielded six separate sets of connections across the hallucinating groups that behaved either similar or differently across groups (Fig. 3B–E). As presented in Fig. 3, a similar pattern of connectivity alterations was observed for schizophrenia patients and non-clinical individuals, including dysconnectivity among fronto-temporal, temporo-temporal, fronto-central and occipital areas (Fig. 3B–E). Bipolar-I disorder patients with hallucinations revealed an inversed pattern of dysconnectivity relative to schizophrenia and non-clinical individuals in terms of showing decreased versus increased connectivity and vice versa. See Supplementary Table 9 for a list of connections per cluster.

### Symptom correlates

None of the clinical variables was associated with either the weighted global efficiency, weighted clustering coefficient, MST leaf fraction, MST diameter or edges within the NBS network in the schizophrenia patients with hallucinations, or non-clinical individuals (all *P* > 0.05, FDR corrected).

### Hallucination modality

When replicating the NBS analysis in a subgroup of bipolar-I participants with only auditory hallucinations, we found similar alterations compared to the total sample of bipolar-I disorder patients with hallucinations, except that increased activity in the occipital cortex was not found in bipolar-I disorder patients with only auditory hallucinations (see Supplementary Fig. 6). When comparing schizophrenia patients with only auditory hallucinations (n = 28) versus healthy controls (n = 228), results were not significant at P > 0.05 FWE corrected.

Using the random forest machine learning algorithm, differentiating between auditory and visual hallucinations in non-clinical individuals was possible with a weighted accuracy of 77.5%, sensitivity of 0.0% and specificity of 93.9%, respectively. The connection between the left superior temporal gyrus and right rolandic operculum (no. 19) was the most important discriminating feature. In both schizophrenia patients and bipolar-I disorder patients, the weighted accuracy was at or below chance-level, meaning than differentiation was not possible (schizophrenia: weighed accuracy of 50.0%, sensitivity of 26.0% and specificity of 64.1%; bipolar-I disorder: weighted accuracy of 28.6%, sensitivity of 67.3% and specificity of 7.0%). See Supplementary Fig. 7 for an overview of the results per group.

## Discussion

This study compared functional connectivity alterations related to hallucinations in a large sample of patients with schizophrenia, bipolar-I disorder, non-clinical individuals, and healthy controls. A range of connections was found to be altered in schizophrenia patients and non-clinical individuals with hallucinations. These alterations involved mainly increased connectivity between the insula, cingulate cortex, auditory, and language-related areas in non-clinical individuals and schizophrenia patients, compared to controls. The non-clinical individuals and schizophrenia patients exhibited remarkably similar disruptions of functional connectivity. In contrast, differential effects were observed for bipolar-I disorder patients with hallucinations versus controls, involving mainly *decreased* connectivity between fronto-temporal and fronto-striatal areas. Bipolar-I disorder patients without hallucinations did not show any connectivity alterations compared to controls. A direct group-wise comparison confirmed connectivity alterations that were similar in patients with schizophrenia and non-clinical individuals, but inversed (decreased versus increased connectivity) in bipolar-I disorder patients. Thus, contrary to our initial hypothesis, we did not find a similar pattern of functional connectivity alterations across the psychosis continuum. This implies that individuals with hallucinations on the psychosis continuum may not necessarily share a neural mechanism, despite overlapping phenomenological features.

Our results are in line with previous findings on schizophrenia and hallucinations, showing alterations in functional connectivity in areas related to sensory processing and cognitive control^4,5,10,12,36–38^. Previous studies also reported increased connectivity among frontal, anterior cingulate, and insular cortex and language-related areas in relation to hallucinatory experiences^11,12^.

Previous findings suggest similar functional connectivity alterations in healthy individuals with hallucinations as observed in schizophrenia with hallucinations^38–40^. Findings of altered structural connectivity in healthy individuals with hallucinations are also broadly consistent with alterations found in schizophrenia with hallucinations^41,42^. Our results confirm previous findings of similar brain alterations in healthy individuals with hallucinations and schizophrenia patients.

Previous studies have showed both similar^6,43^ and dissimilar^44^ connectome alterations in schizophrenia and psychotic bipolar disorder. Our findings could not confirm that bipolar-I patients with hallucinations showed similar connectivity alterations as schizophrenia patients and non-clinical individuals with hallucinations. However, decreased connectivity between frontal and temporal areas, as observed in our sample of bipolar patients with hallucinations, was previously reported in pediatric bipolar disorder patients and related to top-down control^45^. Decreased connectivity between fronto-striatal areas and fronto-temporal areas has also been related to mood dysregulation in bipolar disorder^46,47^.

Our findings should be interpreted in light of methodological considerations. Differences in hallucinatory modality across groups may be associated with the differential findings between schizophrenia patients and non-clinical individuals versus bipolar-I disorder patients with hallucinations. In the latter group, a larger proportion of patients experienced lifetime visual hallucinations (68.9%), with a smaller proportion (52.7%) reporting auditory hallucinations. Previous studies found modality-specific alterations in schizophrenia^48,49^. Indeed, we observed increased connectivity of the visual cortex in patients with bipolar-I disorder patients—but not after repeating the analysis in bipolar-I disorder patients with only auditory hallucinations—suggesting that the involvement of the visual cortex may be linked to the experience of visual hallucinations. To further explore this notion, we applied a random forest machine learning algorithm. Classification accuracies around chance level were found, meaning we did not find a set of connections able to discriminate between effects of auditory versus visual hallucinations. As the sensitivity was around 0% in non-clinical individuals, these results must be interpreted with caution. Because of using our dataset retrospectively, the dataset was not highly suitable to investigate effects of hallucination modality, as the majority of participants endorsed both auditory and visual hallucinations. Future studies could more thoroughly investigate this issue by recruiting patients who experience hallucinations in only one modality.

Furthermore, recent advances have indicated that *structural* atlases might not be best suited to investigate *functional* connectivity, we therefore replicated our results using the functional Power atlas. As the best method regarding the use of brain atlases is currently inconclusive, we included results of both atlases as this may benefit discussion in the field.

By taking advantage of a large cross-diagnostic sample, our findings add important new information on neural mechanism of hallucinations across the psychosis continuum. However, we were unable to include a group of schizophrenia patients *without* lifetime hallucinations, as most schizophrenia patients experience hallucinations in their lifetime. Furthermore, we included participants based on the criterion of having experienced lifetime hallucinations, regardless of current hallucination state. Thus, all participants share a general disposition to hallucinate, but there were differences between groups in current hallucination state. However, using resting state fMRI to study hallucinations is generally thought to be more sensitive to trait-associated connectivity alterations which are relatively stable over time^50,51^. This assertion is in line with our finding that current symptom severity was not significantly correlated with connectivity measures. Moreover, the average frequency of hallucinations in the non-clinical individuals was once per week (much less frequent than in the schizophrenia patients), but these individuals nonetheless exhibited a similar connectivity pattern as the schizophrenia patients. Together, this suggests that our resting state measurements reflect trait- rather than state-characteristics. Moreover, comparison of active hallucination state between schizophrenia and bipolar disorder may be biased by other confounding effects including mood state, as hallucinations in bipolar disorder occur mainly in manic or depressive episodes^17^.

Another limitations concerns the fact that the bipolar-I disorder patients in the current study were all in euthymic phase at time of scanning. Also, the group of bipolar-I disorder patients without hallucinations was smaller in size (n = 42) than the group of bipolar-I disorder patients with hallucinations (n = 74). Taken together, this could have contributed to the null-finding in bipolar-I patients without hallucinations, as larger sample sizes might be needed to pick up more subtle aberrations in this group.

Group-differences in medication use may also have influenced our results. Lithium is suggested to normalize brain function^52–54^, which may explain why bipolar-I disorder patients without lifetime hallucinations did not reveal connectivity differences compared to controls. However, lithium users were evenly balanced across both groups of bipolar-I disorder patients with and without hallucinations, and are thus unlikely to fully explain our results. Also, it is unlikely that our findings can be attributed to the use of antipsychotic medication, as the non-clinical individuals were free of medication use and nevertheless demonstrated similar alterations as the medicated patients with schizophrenia. Confounding factors such as drug-induced hallucinations in the non-clinical group are also unlikely, as none of the non-clinical participants had a positive urine test for illicit drugs^55^.

Potentially confounding effects of age, sex, and motion were tested in several ways, and were found to have minimal influence. Note that our analyses were conducted after stringent exclusion of participants with high motion. Afterward, the relative mean displacement ranged from 0.08 to 0.10 mm across groups, indicating high-quality data.

## Conclusions

The findings of this study suggest that schizophrenia patients and non-clinical individuals with hallucinations exhibit similar alterations in functional connectivity. In contrast, a markedly different pattern of connectivity alterations was observed in bipolar-I patients with a lifetime history of hallucinations, encompassing different regions and involving mainly reductions in connectivity as opposed to increases. These findings suggest a similar neural mechanism for hallucinations in schizophrenia patients and non-clinical individuals, but a different neural mechanism in bipolar-I disorder. If our findings of a different neural mechanism for hallucinations in bipolar disorder can be replicated independently, this would warrant further investigation into whether hallucinations in bipolar-I disorder should be treated differently in clinical practice. Further elucidating the role of large-scale networks in the experience of hallucinations may enable tailored pharmaco-therapeutic interventions designed to restore the balance between these networks.

## Supplementary Information

Supplementary Information.

## Data Availability

The datasets generated during and/or analysed during the current study are not publicly available as this study was part of multiple larger studies of which not all data has yet been analysed and published. Data pertaining to this manuscript are available from the corresponding author on reasonable request.
