# Author Correction: Functional connectome differences in individuals with hallucinations across the psychosis continuum

**DOI:** 10.1038/s41598-021-87849-w

**Published:** 2021-04-14

**Authors:** Maya J. L. Schutte, Marc M. Bohlken, Guusje Collin, Lucija Abramovic, Marco P. M. Boks, Wiepke Cahn, Meenakshi Dauwan, Edwin van Dellen, Neeltje E. M. van Haren, Kenneth Hugdahl, Sanne Koops, René C. W. Mandl, Iris E. C. Sommer

**Affiliations:** 1 Department of Biomedical Sciences of Cells and Systems, University of Groningen, University Medical Center Groningen, Neuroimaging Center, PO Box 196, 9700 AD Groningen, The Netherlands; 2 Department of Psychiatry, University Medical Center Utrecht Brain Center, Utrecht University, Utrecht, The Netherlands; 3 Department of Psychiatry, Beth Israel Deaconess Medical Center and Massachusetts Mental Health Center, Harvard Medical School, Boston, MA USA; 4 McGovern Institute for Brain Research, Department of Brain and Cognitive Sciences, Massachusetts Institute of Technology, Cambridge, MA USA; 5 Department of Intensive Care Medicine and UMC Utrecht Brain Center, University Medical Center Utrecht, Utrecht University, Utrecht, The Netherlands; 6 Department of Child and Adolescent Psychiatry, Erasmus Medical Centre, Rotterdam, The Netherlands; 7 Department of Biological and Medical Psychology, University of Bergen, Bergen, Norway; 8 Department of Psychiatry, Haukeland University Hospital, Bergen, Norway; 9 Department of Radiology, Haukeland University Hospital, Bergen, Norway; 10 NORMENT Center for the Study of Mental Disorders, University of Oslo, Oslo, Norway

Correction to: *Scientific Reports* 10.1038/s41598-020-80657-8, published online 13 January 2021

The Supplementary Information published with this Article contained an error, where an old version of Figure S5 was used.

This error has now been corrected in the Supplementary Information file that accompanies the original Article. The corrected Supplementary Information file is also linked to this correction notice.

## Supplementary Information

Supplementary Information.

