# Supplementary material for: Functional connectome differences in individuals with hallucinations across the psychosis continuum
Source: Sci Rep. 2021 Jan 13;11:1108. doi: 10.1038/s41598-020-80657-8 (PMC7806763; doi:10.1038/s41598-020-80657-8)
Supplement: Supplementary file 1 — Supplementary Information. [file 41598_2020_80657_MOESM1_ESM.pdf]

Supplementary Content

Functional connectome differences in individuals with hallucinations across the psychosis continuum.

Maya J.L. Schutte, Marc M. Bohlken, Guusje Collin, Lucija Abramovic, Marco P.M. Boks, Wiepke Cahn, Meenakshi Dauwan, Edwin van Dellen, Neeltje E.M. van Haren, Kenneth Hugdahl, Sanne Koops, René C.W. Mandl, Iris E.C. Sommer.

Supplementary Methods

Supplementary Results

Supplementary Discussion

Supplementary Table 1 Reasons of exclusion for scans listed per study

Supplementary Table 2 Reasons of exclusion for scans listed per clinical status

Supplementary Table 3 Post-hoc tests of significant differences in age across the groups

Supplementary Table 4 Post-hoc tests of significant differences in sex across the groups

Supplementary Table 5 Overlapping edges between NC-H, SCZ-H and BD-H

Supplementary Figure 1 Distance dependent effects of motion on edge strength

Supplementary Figure 2 Functional connectome alterations in matched subgroups

Supplementary Figure 3 Results of network based statistics using the Power-260 atlas

Supplementary Figure 4 Conjunction analysis on the NBS networks of NC-H, SCZ-H and BD-H

Supplementary Figure 5 Clusters defined by elbow method for k-means clustering

Supplementary Figure 6 Functional connectome disturbances in BD-H and BD-AH.

Supplementary Figure 7 Random forest classification based on the connections of the NBS network.

Supplementary Table 6 Results of the network based statistics for non-clinical individuals with hallucinations versus healthy controls using the AAL atlas

Supplementary Table 7 Results of the network based statistics for schizophrenia patients with hallucinations versus healthy controls using the AAL atlas

Supplementary Table 8 Results of the network based statistics for bipolar-I disorder patients with hallucinations versus healthy controls using the AAL atlas

Supplementary Table 9 Results of the network based statistics across all three hallucination groups using the AAL atlas

Supplementary Table 10 Results of the network based statistics for non-clinical individuals with hallucinations versus healthy controls using the Power atlas.

Supplementary Table 11 Results of the network based statistics for schizophrenia patients with hallucinations versus healthy controls using the Power atlas.

Supplementary Table 12 Results of the network based statistics for bipolar-I disorder patients with hallucinations versus healthy controls using the Power atlas.

Supplementary Table 13 List of features for random forest classifier in non-clinical individuals with hallucinations.

Supplementary Table 14 List of features for random forest classifier in schizophrenia patients with hallucinations.

Supplementary Table 15 List of features for random forest classifier in bipolar-I disorder patients with hallucinations.

Supplementary Table 16 List of abbreviations for brain areas AAL-atlas

Supplementary Methods

Participants

All subjects participated in one of the following studies: *Bipolar Genetics* study (procedures are described by Vreeker and colleagues [1]), *The Outcome of Psychosis and Fitness Therapy* study [2], *Simvastatin for recent onset psychosis* study ([3]; baseline data), *Spectrum* study [4] and the *Understanding Hallucinations* study (inclusion ungoing). This is the first study to combine the resting state functional magnetic resonance imaging (MRI) data across diagnostic categories to investigate a shared neural correlate of hallucinations.

A diagnosis of bipolar-I disorder and a schizophrenia spectrum disorder was defined according to the Diagnostic and Statistical Manual for Mental Disorders (DSM-IV-TR; [5]). Absence or presence of psychopathology was assessed using the Structured Clinical Interview for DSM-IV (SCID-I; [6]), the Mini-International Neuropsychiatric Interview (MINI; [7]), or the Comprehensive Assessment of Symptoms and History Interview (CASH; [8]). The non-clinical individuals did not fulfil criteria of a DSM-IV diagnosis, but did score higher on the Schizotypal Personality Questionnaire (SPQ; [9]) compared to healthy controls [10]. A history of hallucinations and/or delusions was assessed using section B of the SCID-I or CASH interview. All participants were at least 18 years of age. Handedness, sex, and current use of antipsychotic medication and/or lithium were recorded. All participants provided written informed consent. The studies were approved by the Institutional Review Board of the University Medical Center Utrecht and conducted in accordance with the Declaration of Helsinki.

The *Bipolar Genetics* study ([1]; BiG) investigates genetic and phenotypic characteristics of patients with bipolar disorder type I (BD-I) first degree relatives and controls, and is part of a collaboration between the University of California Los Angeles (UCLA), University Medical Center Utrecht (UMCU) GGZ Altrecht, and GGZ InGeest, University Medical Center Groningen, Delta Center for Mental Health Care, Dimence, Psychiatric Institute The Hague (PsyQ) and Reinier van Arkel Group. For the current study, we included 185 patients with a bipolar-I disorder, and 135 healthy controls recruited via the BiG study.

The Outcome of Psychosis and Fitness Therapy study ([2]; TOPFIT) is a multicenter study investigating physical health and the effects of physical therapy in patients with schizophrenia spectrum disorder and healthy controls. Patients were recruited at several Dutch mental health care institutes, including the University Medical Center Utrecht, GGZ Altrecht, GGZ Duin- en Bollenstreek, and GGZ Friesland. For the current study, we included 36 patients with a schizophrenia spectrum disorder, and 42 healthy controls recruited for the TOPFIT study.

The *Simvastatin for recent onset psychosis* study [3] is an ongoing double-blind placebo controlled medication trial that investigates add-on treatment of simvastatin in patients with a recent onset psychosis. Patients were recruited from several Dutch mental healthcare institutes, including the University Medical Center Utrecht, University Medical Center Groningen, GGZ Altrecht, GGZ InGeest, Academic Medical Center Amsterdam, GGZ Arkin. The inclusion criteria were a) between 18-50 years; b) a diagnosis of psychosis not otherwise specified (NOS), schizophrenia, schizophreniform disorder, schizotypical personality disorder; c) the first psychotic episode was not longer than 3 years ago. For the current study, we included 11 patients with a schizophrenia spectrum disorder recruited via the Simvastatin study.

The *Spectrum* study [4] investigated the effect of hallucinations in schizophrenia patients and participants with non-clinical hallucinations. Participants were recruited via a website www.verkenuwgeest.nl ("explore your mind"). Inclusion criteria for non-clinical voice hearers were: a) voices were distinct from thoughts and had a "hearing" quality; b) voices were experienced at least once a month; c) absence of psychiatric disorders other than anxiety or depressive disorder in full remission as assessed in a psychiatric examination using the CASH, and the SCID-II interviews; d) absence of alcohol or drug abuse for at least 3 months prior to the assessment; e) absence of chronic somatic disorder. In the current study, we included 51 healthy controls, 40 non-clinical individuals with hallucinations, and 37 patients with a schizophrenia spectrum disorder.

The *Understanding Hallucinations* (UH) study is an ongoing multicenter cross-sectional study that investigates phenomenology and underlying brain mechanisms of hallucinations across a wide range of disorders (clinicaltrials.gov identifier NCT02460965). Participants were recruited via several institutes in the Netherlands, including the University Medical Center Utrecht, Parnassia Psychiatric Institute, VU medical center Amsterdam, Diakonessenhuis Utrecht, and University Medical Center Groningen. Inclusion criteria were; 1) age ≥ 18 years; and 2) experienced hallucinations in the past month; or never experiences hallucinations in life. For the current study, we included 15 patients with a schizophrenia spectrum disorder who were recruited for the Understanding Hallucinations study.

Data acquisition
All participants were acquired on the same 3 tesla Achieva Philips clinical MRI scanner equipped with an 8-channel SENSE head coil at the University Medical Center Utrecht (Philips, Best, The Netherlands). Anatomical 3D high-resolution T1-weighted images were obtained for anatomical reference with the following parameter settings: echo time [TE] = 4.6 ms, repetition time [TR] = 10 ms, flip angle = 90º, Field of View (FOV) = 240 mm/100%, voxel size 0.75 × 0.75 × 0.80 mm, reconstruction matrix = 200 x 320 x 320. For 49 participants included in the Spectrum study [4], T1-weighted images with a lower resolution were available: 160 contiguous sagittal slices (TE = 4.6 ms, TR = 10 ms, flip angle = 8°, FOV = 224 mm, 1 × 1 × 1 mm voxels). Task-free resting state functional data were acquired using a 3D PRESTO sequence that allowed for fast functional brain coverage every 609 ms [11]. All participants were instructed to keep their eyes closed. Parameters settings were: (40 (coronal) slices, TE = 32.4 ms, TR = 21.75 ms, flip angle = 10°, FOV = 224 x 256 x 160, 4 x 4 x 4 mm voxels). Using this sequence, between 600-1000 images were obtained depending on the study for which the data was acquired, after which all resting state scans were resized to the first 600 images (~6 minutes). All scans were checked for radiological abnormalities.

Data preprocessing
Preprocessing was done using the FMRIB Software Library (FSL) version 5.0.4 [12] with FEAT's default settings, including skull stripping (BET), motion correction with MCFLIRT, spatial smoothing (5mm kernel at full width at half maximum) and high-pass filtering (100-second cut-off). The mean global signal was not included, as this could potentially bias group differences or increase the risk of removing a valuable signal [13-15]. In-scanner motion can influence functional connectivity measures and is known to disproportionally affect the functional connectome of patients versus controls [16-17]. Several steps were taken to prevent a systematic motion-related bias in this study following recent developments in the literature. If the relative root mean square displacement over all frames exceeded 0.2mm; or if 20 individual frames exceeded the threshold of 0.25mm, these subjects were excluded from further analysis [18]. ICA-AROMA was subsequently applied for denoising [19-20]. More information on exclusion of scans due to missing clinical data, poor imaging, data quality, processing errors, motion artefacts, or brain abnormalities reported by the radiologist can be found in the Supplementary material.

Functional network reconstruction
The average time series for each participant were extracted from N=90 functional regions (nodes) of the automated anatomical labeling (AAL) atlas [21] and N=260 nodes of the Power atlas [22] covering the whole brain, including cortical and subcortical regions. We applied wavelet decomposition on the raw time series to extract a signal in the frequency domain of 0.05 - 0.1 Hz (Scale 4), as resting state studies commonly apply bandpass filtering over the frequency domain between 0.01 - 0.1 Hz [23-25]. Wavelet-based methods have several advantages in terms of denoising [26], robustness to outliers [27], autocorrelations and has the advantage of bypassing the role of positive and negative edge weights compared the traditional band-pass filtering and Pearson correlation coefficients [28]. Because a coherence value is always between 0 and 1, this facilitates possible problems of how to deal with negative edge weights. The coherence was calculated as the mean squared coherence between time series over the chosen wavelet scale in Matlab, using the *mscohere* function.

Global connectome disturbances
We analyzed disturbances in global network organization related to hallucinations. Two key properties were investigated to characterize network topology. The first property is the weighted global efficiency, which provides information on overall communication efficiency throughout the network [29] and is hence a measure of network integration. It was calculated as the average inverse shortest path length between all node pairs [29]. The second property is the weighted clustering coefficient, which reflects the degree to which nodes in the network tend to cluster together, and is a metric of network segregation. It was calculated as the weighted level of functional connectivity that exists between each nodes' neighbours, proportional to the maximum degree of connectivity [29]. The graph metrics were computed using the Brain Connectivity Toolbox [29]. Thresholding connectivity matrices can bias graph metrics due to differences in edge density and functional connectivity strength between patient and control groups [30-31]. We therefore used unthresholded connectivity matrices and tested if the connectivity strength differed across groups.

Minimum Spanning Tree (MST) analysis provides information on a network's topology and is therefore insensitive to connection strength [32-33]. The Minimum Spanning Tree (MST) is a subnetwork of the original weighted connectivity matrix, connecting all nodes in the network without forming loops, and therefore represents the strongest connections in the network (e.g. a maximum spanning tree) [32]. For each subject, the MST network was constructed by consecutively adding connections with the highest-ranking weight. When adding a new connection resulted in the formation of a loop, this connection was omitted and the next connection in order of weight was added

to the network. This procedure was repeated until all nodes were connected, resulting in a unique MST for each subject with a fixed number of (N) 90 nodes and (M = N-1) 89 links. Hence, no bias was induced due to differences in connection density or strength between subjects. The MST therefore represents the strongest connections in the network (e.g. a maximum spanning tree), and network topology was compared using the MST leaf fraction and MST diameter [32].

A combination of two MST measures, namely the diameter and the leaf fraction can be used to determine if the network's topology is more line-like (i.e., less integrated) or more star-like (i.e., more integrated) network [32]. The MST diameter is calculated as the longest distance (in number of edges) between any two nodes of the MST. The MST leaf fraction, reflecting the number of nodes that only link to one other node (i.e., degree 1). A longer diameter and lower leaf number will reflect a more line-like network, whereas a more integrated star-like network will have a shorter diameter and larger leaf number [32].

K-means clustering

We computed an averaged connectivity matrix per participant group and subtracted the average matrix of healthy controls to obtain three difference matrices. These differences matrices were converted into three vectors, thereby only extracting those connections that were part of the NBS component. The three vectors were entered into a k-means clustering algorithm.

The k-means clustering method is an unsupervised machine learning algorithm that groups data into a pre-specified number of k clusters. We determined the optimal number of according to Madhulatha and colleagues [34], by running the k-means clustering algorithm for k range of values (e.g. 1-10 clusters) and subsequently calculating the sum of squared errors (SSE) at each k. The most favorable balance between a low SSE and low k determines the optimum number of clusters [34]. In this study, the elbow method validated an optimum number of six clusters (Supplementary Figure 4).

Effects of age, sex, motion and modality

Due to the relatively large differences in age and sex between the groups, we tested for possible collinearity. To identify collinearity among variables the variance inflation factor (VIF) was calculated in SPSS 22.0 using linear regression. As a rule of thumb, a VIF > 3 warrants extra checking, and a VIF > 5 means that collinearity is highly likely. To further explore possible effects of age and sex as covariates, we replicated the Network Based Statistics analyses in smaller matched subgroups (without including age and sex as covariates). These smaller subgroups were matched using exact case-control matching in SPSS 20.0.

The residual relationship between subject movement and connectivity was evaluated according to two benchmarks; 1) assess residual relationship between motion and connectivity by estimating the residual QC-FC (quality control / functional connectivity) correlation; 2) estimating distance-dependent effects of motion on connectivity. The QC-FC relationship was computed by correlating the network edge weight of the 90-nodes with the mean relative displacement motion as calculated by MCFLIRT function in FSL [35]. To account for the participant's age and sex, a partial correlation was calculated. This was subsequently used to calculate the number of edges that significantly correlated with subject motion after using the false discovery rate (FDR) [36]. Secondly, distance dependent effects were estimated by correlating the Euclidean distance between the center of each node with the QC-FC correlation. Additional data on the residual relation between motion and functional connectivity is provided in Supplementary Figure 1.

Random forest classification algorithm

The random forest classifier has the advantage that cross-validation is done internally, meaning that no separate test set of participants is needed to estimate the generalized error of the training set [39]. The cross-validation is done internally by out-of-bag (OOB) error estimate. In each bootstrap training sample, one-third of the features are left out and *not* used in assembling the decision tree. As the random forest is built, each tree is tested on features not used in building that tree, termed OOB examples. Each feature that is left out is put down the tree to obtain a classification. At the end of the run, the model predicts the class that received most of the votes every time feature x was OOB, as well as the proportion of time the class is not equal to the original class of feature x. This is averaged over all features resulting in the OOB error estimate [39].

Each feature per classification receives a variable importance (VIMP) score between 0 and 1. This score is computed by the mean decrease in the Gini impurity criterion. This Gini impurity specifies how often a specific feature was selected for a split, and how large its overall discriminative value was for the classification. By adding up the Gini impurity decreases for each feature across all trees in the random forest, the feature importance (i.e. variable) is obtained.

The classification metrics sensitivity, specificity, and accuracy were determined as follows:

		**True Class**	
		**Positive**	**Negative**
**Predicted class**	**Positive**	True Positive TP	False Positive FP
	**Negative**	False Negative FN	True Negative TN

Sensitivity	Specificity	Accuracy
$\frac{TP}{TP+FN}$	$\frac{TN}{FP + TN}$	$\frac{TP + TN}{TP+FP+FN+TN}$

For a more complete description of the classification algorithm, see Dauwan and colleagues [36]. The weighted accuracy was calculated according to Chen and colleagues [37] to correct for unequal sample sizes within each classification.

Supplementary Results

Subjects

Of all scans collected in the five studies, a subset of 749 participants was selected based on presence of a resting state and anatomical scan. Of these, n = 196 participants were excluded due to missing clinical data, poor imaging, data quality, processing errors, effects of motion, or brain abnormalities reported by the radiologist. See Supplementary Table 1 for a detailed overview of excluded scans per study, and Supplementary Table 2 for an overview of excluded scans per clinical status (e.g. NC-H, SCZ, BD, CTRL). Next, we selected only those participants who experienced lifetime hallucinations, and bipolar patients without any lifetime history of psychotic experiences. We therefore excluded another 69 patients with bipolar-I disorder, and 6 patients with schizophrenia. Exclusion of these scans resulted in a final eligible pool of n = 483 subjects. On average, sex and age differed across the participant groups. Tukey post-hoc tests were conducted to provide more information on the exact differences (see Supplementary Table 3 for post-hoc tests on age, and Supplementary Table 4 for post-hoc tests on sex). Because age and sex differed across the groups, these variables were entered as covariates in the subsequent analyses.

Effects of age and sex

No significant relationship was present between age and sex was $\tau = -0.055$, $P = 0.145$. The collinearity diagnostics between age and sex was VIF = 1.000; tolerance = 1.000, indicating a low possibility of collinearity (VIF < 3). Age and sex were thus entered as covariates in the analysis. To further explore the effects of age and sex, we also replicated the NBS analyses in smaller matched subgroups, see Supplementary Fig 2. A similar pattern of connectivity disturbances was demonstrated for the non-clinical individuals and patients with schizophrenia. The analysis for patients with bipolar disorder-I was not significant, although a trend was observed ($P = 0.097$). This is probably due to a power problem as this analysis was done in a smaller matched subgroup. We therefore repeated this analysis at a more lenient significance level ($P < 0.10$) which yielded a similar pattern of connectivity disturbances as in the original analyses. The bipolar patients without hallucinations did not show any significant differences with healthy controls, neither at a more lenient significance threshold ($P > 0.10$).

Effects of motion
The residual relationship between motion and connectivity strength was estimated, revealing that none of the edges was significantly related to motion after FDR correction ($P < 0.001$, for all tests). The median QC-FC for healthy controls was 0.07; for patients with schizophrenia 0.06; for patients with bipolar disorder 0.05; and for non-clinical individuals 0.11. These numbers are in correspondence with previous findings on ICA-AROMA by Ciric and colleagues [35]. Distance dependent effects of motion are represented by the correlation displayed in each of the graphs in Supplementary Figure 1. The distance dependent effects. The results demonstrate that the distance dependent effect on motion was minimal for all groups.

Global connectome disturbances
The groups did not differ in functional connectivity strength (F (4) = 1.0; $P = 0.434$), meaning its effects on graph theoretical measures can be considered minimal [30]. None of the hallucination groups differed in terms of global network organization relative to healthy controls, neither in graph theoretical or MST measures ($P > 0.05$, FDR corrected).

Network Based Statistics
The NBS contrasts schizophrenia patients vs healthy controls, and non-clinical individuals vs healthy controls were both tested using a F-test at T = 13.0; $P < 0.05$ at 10,000 permutations. The contrast bipolar-I disorder patients with hallucinations vs healthy controls was tested at T = 8.5; $p < 0.05$ at 10,000 permutations. Differences between bipolar-I disorder patients vs healthy controls was tested at a range of T-thresholds, but did not result in any significant differences at either of the thresholds.

According to Zalesky and colleagues [40], the choice of the T-statistic is somewhat arbitrary, but does not affect the specificity of the results. We therefore choose T-statistics that provided a roughly equal number of connections across the contrasts to facilitate visual comparison of results. All significant connections in the NBS network are displayed in Supplementary Table 6 for patients with bipolar disorder, Supplementary Table 7 for patients with schizophrenia, and Supplementary Table 8 for non-clinical individuals with hallucinations. The results of the overall group comparison of all hallucinating individuals compared to healthy controls are displayed in Supplementary Table 9.

Conjunction analysis
To test for precise overlap of connections in each of the NBS networks, a conjunction analysis was applied. The connections in the NBS networks of the non-clinical individuals and schizophrenia patients were found to overlap with eight connections, while the non-clinical individuals and schizophrenia patients showed overlap with bipolar-I patients with hallucinations in only one connection (see Supplemental Figure 4 and Supplemental Table 5).

Supplementary Discussion

Implication for future research
Connectivity disturbances in areas such as the cingulate gyrus, insula, hippocampal complex, precuneus and language areas suggests involvement of several large-scale networks, including the auditory, attention and task-control networks in the experience of hallucinations. This typical pattern could suggest that sensory areas may not be adequately mediated by higher-order cognitive regions [41]. These findings can be interpreted in light of the bottom-up/top-down explanatory model which proposes that hallucinations may arise from an imbalance between sensory input (bottom-up) and top-down inhibitory control on sensory areas [42-45]. Disruption of bottom-up processing networks could lead to disturbed interpretation of incoming sensory input, causing patients to hallucinate.

For example, the circular inference theory explains the experience of hallucinations in terms of an imbalance between bottom-up and top-down information [46]. Reduced inhibitory control is hypothesized to lead to top-down and bottom-up information being reverberated (i.e. prior beliefs are misinterpreted as sensory observations) [46]. This may fit with the observed increased connectivity between the frontal and temporal area in both schizophrenia patients and non-clinical individuals in this study, as multiple overcounting of the same redundant sensory and prior information means that both areas will be more active at the same time (e.g. resulting in a higher correlation of BOLD signals) than to be expected in controls.

In support of the reduced inhibitory control theory, previous studies demonstrated that patients with schizophrenia have difficulties to suppress irrelevant information as established by various cognitive tasks measuring inhibitory control [47-50]. Similar deficits in inhibitory control were reported in non-clinical individuals with hallucinations [51-52]. Reduced inhibitory control was previously reported for bipolar patients in a manic episode

[53], but to a lesser extent in euthymic states [54]. The bipolar-I disorder patients in the current study were all in euthymic phase at time of scanning. Euthymic bipolar patients have been found to perform intermediate to schizophrenia patients and controls on executive functioning tasks [55]. In all, these findings might point to mild inhibitory problems related to hallucinations, albeit to a lesser extent in bipolar-I disorder patients than schizophrenia patients and non-clinical individuals with hallucinations.

The present findings are correlational, and can therefore not confirm our hypothesis that connectivity between frontal, parietal and cingulate areas relates to inhibitory dysfunction. Future studies could investigate whether these disturbed connections point to inhibitory and excitatory dysfunction by studying global glutamate (Glu) receptor hypofunction (or the N-methyl-d-aspartate receptor (NMDA-R)) and gamma-aminobutyric acid (GABA) transmission related to dysconnectivity profiles [42, 46, 56]. This could for example be done by combining fMRI with 1H-MR spectroscopy (MRS) to measure levels of Glu and GABA functioning in particular areas of the cortex [57].

References

1. Vreeker, A., *et al.* High educational performance is a distinctive feature of bipolar disorder: a study on cognition in bipolar disorder, schizophrenia patients, relatives and controls. *Psychol Med.* **46**, 807-18 (2016).
2. Scheewe, T.W., *et al.* Exercise therapy, cardiorespiratory fitness and their effect on brain volumes: a randomised controlled trial in patients with schizophrenia and healthy controls. *Eur Neuropsychopharmacol.* **23**, 675-85 (2013).
3. Begemann, M.J., *et al.* Simvastatin augmentation for recent-onset psychotic disorder: A study protocol. *BBA Clin.* **4**, 52-58 (2015).
4. Sommer, I.E., *et al.* Healthy individuals with auditory verbal hallucinations; who are they? Psychiatric assessments of a selected sample of 103 subjects. *Schizophr Bull.* **36**, 633–41 (2010).
5. APA. *Diagnostic and Statistical Manual of Mental* Disorders (4th ed. Text Revision). Washington. (American Psychiatric Association, 2000).
6. First, M., Spitzer, R., Gibbon, M., Williams, J. *Structured Clinical Interview for DSM-IV-TR Axis I Disorders, Research Version, Patient Edition.* (SCID-I/P). New York: Biometrics Research. (New York State Psychiatric Institute; 2002)
7. Lecrubier, Y., *et al.* The Mini International Neuropsychiatric Interview (M.I.N.I.): a short diagnostic structured interview: reliability and validity according to the CIDI. *European Psychiatry.* **12**, 224-231 (1997).
8. Andreasen, N.C., Flaum, M., Arndt, S. The Comprehensive Assessment of Symptoms and History (CASH). An instrument for assessing diagnosis and psychopathology. *Arch Gen Psychiatry.* **49**, 615-23 (1992).
9. Raine, A. The SPQ: a scale for the assessment of schizotypal personality based on DSM-III-R criteria. *Schizophr Bull.* **17**, 555-64 (1991).
10. van Lutterveld, R., *et al.* Cortical thickness in individuals with non-clinical and clinical psychotic symptoms. *Brain.* **137**, 2664-9 (2014).
11. Neggers, S.F., Hermans, E.J., Ramsey, N.F. Enhanced sensitivity with fast three-dimensional blood-oxygen-level-dependent functional MRI: Comparison of SENSE-PRESTO and 2D-EPI at 3T. *NMR Biomed.* **21**, 663–676 (2008).
12. Smith, S.M., *et al.* Advances in functional and structural MR image analysis and implementation as FSL. *NeuroImage.* **23**, 208-19 (2004).
13. Saad, Z.S., *et al.* Trouble at rest: how correlation patterns and group differences become distorted after global signal regression. *Brain connectivity.* **2**, 25-32 (2012).
14. Gotts, S.J., *et al.* The perils of global signal regression for group comparisons: a case study of Autism Spectrum Disorders. *Front. Human. Neurosci.* **12**, 7-356 (2013).
15. Yang, G.J., *et al.* Altered global brain signal in schizophrenia. *Proc. Natl. Acad. Sci. U. S. Am.* **111**, 7438–7443 (2014).
16. Power, J.D., Barnes, K.A., Snyder, A.Z., Schlagger, B.L., Petersen, S.E. Spurious but systematic correlations in functional connectivity MRI networks arise from subject motion. *Neuroimage.* **59**, 2142-2154 (2012).
17. van Dijk, K.R.A, Sabuncu, M.R., Buckner, R.L. The influence of head motion on intrinsic functional MRI. *Neuroimage.* **59**, 431-438 (2012).
18. Satterthwaite, T.D., *et al.* Impact of in-scanner head motion on multiple measures of functional connectivity: relevance for studies of neurodevelopment in youth. *Neuroimage.* **60**, 623-32 (2012).

19. Pruim, R.H., Mennes, M, Buitelaar, J.K., Beckmann, C.F. Evaluation of ICA-AROMA and alternative strategies for motion artifact removal in resting state fMRI. *Neuroimage.* **112**, 278-87 (2015).
20. Pruim, R.H., *et al.* ICA-AROMA: A robust ICA-based strategy for removing motion artifacts from fMRI data. *Neuroimage.* **112**, 267-77 (2015).
21. Tzourio-Mazoyer, B., *et al.* Automated anatomical labeling of activations in SPM using a macroscopic anatomical parcellation of the MNI MRI single-subject brain. *Neuroimage*. **15**, 273–289 (2002).
22. Power, J.D., *et al*. Functional network organization of the human brain. *Neuron*. **72**, 665-678 (2011).
23. Percival, D.B., Walden, A.T. *Wavelet Methods for Time Series Analysis.* (Cambridge University Press; 2000).
24. Grinsted, A., Moore, J.C., Jevrejeva, S. Application of the cross wavelet transform and wavelet coherence to geophysical time series. *Nonlin Processes Geophys*. **11**, 561-566 (2004).
25. Zhang, Z., Telesford, Q.K., Giusti, C., Lim, K.O., Bassett, D.S. Choosing Wavelet Methods, Filters, and Lengths for Functional Brain Network Construction. *PLoS One.* **11**, e0157243 (2016).
26. Fadili, M.J. & Bullmore, E.T. A comparative evaluation of wavelet-based methods for hypothesis testing of brain activation maps. *Neuroimage.* **23**,1112-1128 (2004).
27. Archard, S., Salvador, R., Whitcher, B., Suckling, J., Bullmore, E. A resilient low-frequency, small-world human brain functional network with highly connected association cortical hubs. *J Neurosci.* **26**, 63-72 (2006).
28. Gu, S., *et al.* Emergence of system roles in normative neurodevelopment. *Proc Natl Acad Sci USA.* **112**, 13681-6 (2015).
29. Onnela, J.P., Saramäki, J., Kertész, J., Kaski, K. Intensity and coherence of motifs in weighted complex networks. *Phys Rev E Stat Nonlin Soft Matter Phys.* **71**, 065103 (2005).
30. van Wijk, B.C., Stam, C.J., Daffertshofer, A. Comparing brain networks of different size and connectivity density using graph theory. *PLoS One*. **5**, e13701 (2010).
31. van den Heuvel, M.P., *et al*. Proportional thresholding in resting-state fMRI functional connectivity networks and consequences for patient-control connectome studies: Issues and recommendations. *Neuroimage*. **152**, 437-449 (2017).
32. Stam, C.J., *et al.* The trees and the forest: Characterization of complex brain networks with minimum spanning trees. *Int J Psychophysiol*. **92**,129-38 (2014).
33. Tewarie, P., van Dellen, E., Hillebrand, A., Stam, C.J. The minimum spanning tree: an unbiased method for brain network analysis. *Neuroimage.* **104**, 177-88 (2015).
34. Madhulatha, T.S. An Overview on Clustering Methods. *IOSR Journal of Engineering*. **2**, 719-725 (2012).
35. Jenkinson, M., Bannister, P., Brady, J.M., Smith, S.M. Improved Optimisation for the Robust and Accurate Linear Registration and Motion Correction of Brain Images. *NeuroImage.* **17**, 825-841 (2002).
36. Ciric, R., *et al.* Benchmarking of participant-level confound regression strategies for the control of motion artefact in studies of functional connectivity. *Neuroimage.* 154, 174-187 (2017).
37. Dauwan, M., *et al.* Random forest to differentiate dementia with Lewy bodies from Alzheimer's disease. *Alzheimers Dement (Amst).* **19**, 99-106 (2016).
38. Chen, C., Liaw, A., Breiman, L. Using random forest to learn imbalanced data. Dept. Statistics, Univ. California, Berkeley, CA, *Tech. Rep.* **666** (2004).
39. Breiman L, University of California Random forest. *Mach Learn.* **45**, 1–35 (1999).
40. Zalesky, A., Cocchi, L., Fornito, A., Murray, M.M., Bullmore, E. Connectivity differences in brain networks. *Neuroimage.* **60**, 1055-62 (2012).
41. Hugdahl, K. "Hearing voices": Auditory hallucinations as failure of top-down control of bottom-up perceptual processes. *Scandinavian Journal of Psychology.* **50**, 553-560 (2009).
42. Friston, K.J. Models of brain function in neuroimaging. *Annu. Rev. Psychol.* **56**, 57-87 (2005).
43. Fletcher, P.C., Frith, C.D. Perceiving is believing: a Bayesian approach to explaining the positive symptoms of schizophrenia. *Nat Rev Neurosci.* **10**, 48-58 (2009).
44. Powers, A.R., Gancsos, M.G., Finn, E.S., Morgan, P.T., Corlett, P.R. Ketamine-induced hallucinations. *Psychopathology.* **48**, 376–385 (2015).
45. O'Callaghan, C., Kveraga, K., Shine, J.M., Adams, R.B. Jr., Bar, M. Predictions penetrate perception: Converging insights from brain, behaviour and disorder. *Conscious Cogn.* **47**, 63-74 (2017).
46. Jardri, R., Denève, S. Circular inferences in schizophrenia. *Brain.* **136**, 3227-41 (2013).
47. Waters, F.A., Badcock, J.C., Maybery, M.T., Michie, P.T. Inhibition in schizophrenia: association with auditory hallucinations. *Schizophr Res.* **62**, 275-80 (2003).
48. Badcock, J.C., Mahfouda, S., Maybery, M.T. Hallucinations and inhibitory functioning in healthy young adults with high and low levels of hypomanic personality traits. *Cogn Neuropsychiatry*. **20**, 254-69 (2015).

49. Soriano, M.F., Jiménez, J.F., Román, P., Bajo, M.T. Intentional inhibition in memory and hallucinations: directed forgetting and updating. *Neuropsychology.* **23**, 61–70 (2009).
50. Hugdahl, K., *et al.* Failure of attention focus and cognitive control in schizophrenia patients with auditory verbal hallucinations: evidence from dichotic listening. *Schizophr Res.* **147**, 301–309 (2013).
51. Paulik, G., Badcock, J.C., Maybery, M.T. Poor intentional inhibition in individuals predisposed to hallucinations. *Cogn Neuropsychiatry.* **12**, 457-70 (2007).
52. Daalman K, *et al.* Auditory verbal hallucinations and cognitive functioning in healthy individuals. *Schizophr Res.* **132**, 203-7 (2011).
53. Badcock, J.C., Hugdahl, K. Cognitive mechanisms of auditory verbal hallucinations in psychotic and non-psychotic groups. *Neurosci Biobehav Rev.* **36**, 431-8 (2012).
54. McGrath, J., Scheldt, S., Clair, A. Performance on tests sensitive to impaired executive ability in schizophrenia, mania, and well controls: Acute and subacute phases. *Schizophr Res.* **26**, 127–137 (1997).
55. Larson, E.R., Shear, P.K., Krikorian, R., Welge, J., Strakowski, S.M. Working memory and inhibitory control among manic and euthymic patients with bipolar disorder. *J Int Neuropsychol Soc.* **11**, 163-72 (2005).
56. Corlett, P.R., Frith, C.D., Fletcher, P.C. From drugs to deprivation: a Bayesian framework for understanding models of psychosis. *Psychopharmacology (Berl).* **206**, 515-30 (2009).
57. Hugdahl, K., *et al.* Glutamate as a mediating transmitter for auditory hallucinations in schizophrenia: a (1)H MRS study. *Schizophr Res.* **161**, 252-60 (2015).
58. Tzourio-Mazoyer, B., *et al*. Automated anatomical labeling of activations in SPM using a macroscopic anatomical parcellation of the MNI MRI single-subject brain. Neuroimage. **15**, 273–289 (2002).

Supplementary Table 1. Reasons of exclusion for scans listed per study[a].

	Studies				
**Reason for exclusion**	**Spectrum**	**BIG**	**TOPFIT**	**Simvastatin**	**UH**
No data on lifetime hallucinations	14	4	3	2	1
Radiological exclusions	2	8	0	0	0
Preprocessing errors	9	67	13	1	1
Motion outliers	27	34	7	2	1
Total	*52*	*113*	*23*	*5*	*3*

[a]The total number of selected scans per study should be taken into account when interpreting these numbers, which are Spectrum n = 133, BIG n = 320, TOPFIT n = 78, Simvastatin n = 11, UH n = 16. Abbreviations: BIG Bipolar Genetics study; TOPFIT The Outcome of Psychosis and Fitness Therapy study; UH Understanding Hallucinations.

Supplementary Table 2. Reasons of exclusion for scans listed per clinical status[a].

	Clinical status			
**Reason for exclusion**	**SCZ**	**BD-I**	**NC-H**	**HC**
No data on lifetime hallucinations	11	0	1	5
Radiological exclusions	1	5	1	2
Preprocessing errors	13	38	1	36
Motion outliers	29	29	5	8
Total	*54*	*72*	*8*	*51*
Age, Mean (SD), y	35.2 (11.4)	50.26 (12.7)	45.7 (9.9)	41.0 (17.3)

[a]Number of cases with missing clinical status n=11. Number of cases with missing data on age: SCZ-H n = 4; NC-H n = 1;. Abbreviations: SCZ-H schizophrenia with hallucinations; BD-I bipolar-I disorder with and without hallucinations; NC-H non-clinical individuals with hallucinations; HC healthy controls.

Supplementary Table 3. Post-hoc tests of significant differences in age across the groups[a].

Participants		Mean difference	*P* value
HC	NC-H	-2.4	.827
	SCZ-H	8.4	.000*
	BD-H	-5.6	.014*
	BD	-11.6	.000
NC-H	HC	2.4	.827
	SCZ-H	10.8	.000*
	BD-H	-3.2	.733
	BD	-9.2	.015*
SCZ-H	HC	-8.4	.000*
	NC-H	-10.8	.000*
	BD-H	-14.0	.000*
	BD	-20.0	.000*
BD-H	HC	5.6	.014*
	NC-H	3.2	.733
	SCZ-H	14.0	.000*
	BD	-6.0	.129
BD	HC	11.6	.000*
	NC-H	9.2	.015*
	SCZ-H	20.0	.000*
	BD-H	6.0	.129

[a]Differences are deemed significant at $p < 0.05$ (*indicated with asterisk). Post-hoc tests done using Tukey HSD test. Abbreviations: NC-H individuals with non-clinical hallucinations; BD-H bipolar disorder with lifetime history of hallucinations; BD bipolar disorder without lifetime history of hallucinations; SCZ-H schizophrenia spectrum disorder with hallucinations.

Supplementary Table 4. Post-hoc tests of significant differences in sex across the groups[a].

Participants		Chi-square	*P* value
HC	NC-H	10.7	.001*
	SCZ-H	6.0	.014*
	BD-H	0.2	.646
	BD	.04	.840
NC-H	HC	10.7	.001*
	SCZ-H	21.0	<.001*
	BD-H	8.7	.010*
	BD	7.5	.006*
SCZ-H	HC	6.0	.014*
	NC-H	21.0	<.001*
	BD-H	5.5	.019*
	BD	2.1	.144
BD-H	HC	0.2	.646
	NC-H	8.7	.010*
	SCZ-H	5.5	.019*
	BD	0.2	.622
BD	HC	0.0	.840
	NC-H	7.5	.006*
	SCZ-H	2.1	.144
	BD-H	0.2	.622

[a]Differences are deemed significant at $p < 0.05$ (*indicated with asterisk). Post-hoc tests done using Chi-square tests. Abbreviations: NC-H individuals with non-clinical hallucinations; BD-H bipolar disorder with lifetime history of hallucinations; BD bipolar disorder without lifetime history of hallucinations; SCZ-H schizophrenia spectrum disorder with hallucinations.

Supplementary Table 5. Overlapping edges between NC-H, SCZ-H, and BD-H.

Diagnoses	Region	Region
NC-H and BD-H	Cuneus L	Lingual R
NC-H and SCZ-H	Frontal Inf Oper R	Supramarginal R
	Insula R	Supramarginal R
	Rolandic Oper R	Supramarginal R
	Insula L	Temporal Sup L
	Rolandic Oper R	Temporal Sup L
	Rolandic Oper R	Temporal Mid L
	Rolandic Oper R	Temporal Mid R
SCZ-H and BD-H	Rolandic Oper R	Temporal Inf R

Note: abbreviations: SCZ-H schizophrenia with hallucinations; BD-H bipolar-I disorder with hallucinations; NC-H non-clinical individuals with hallucinations.

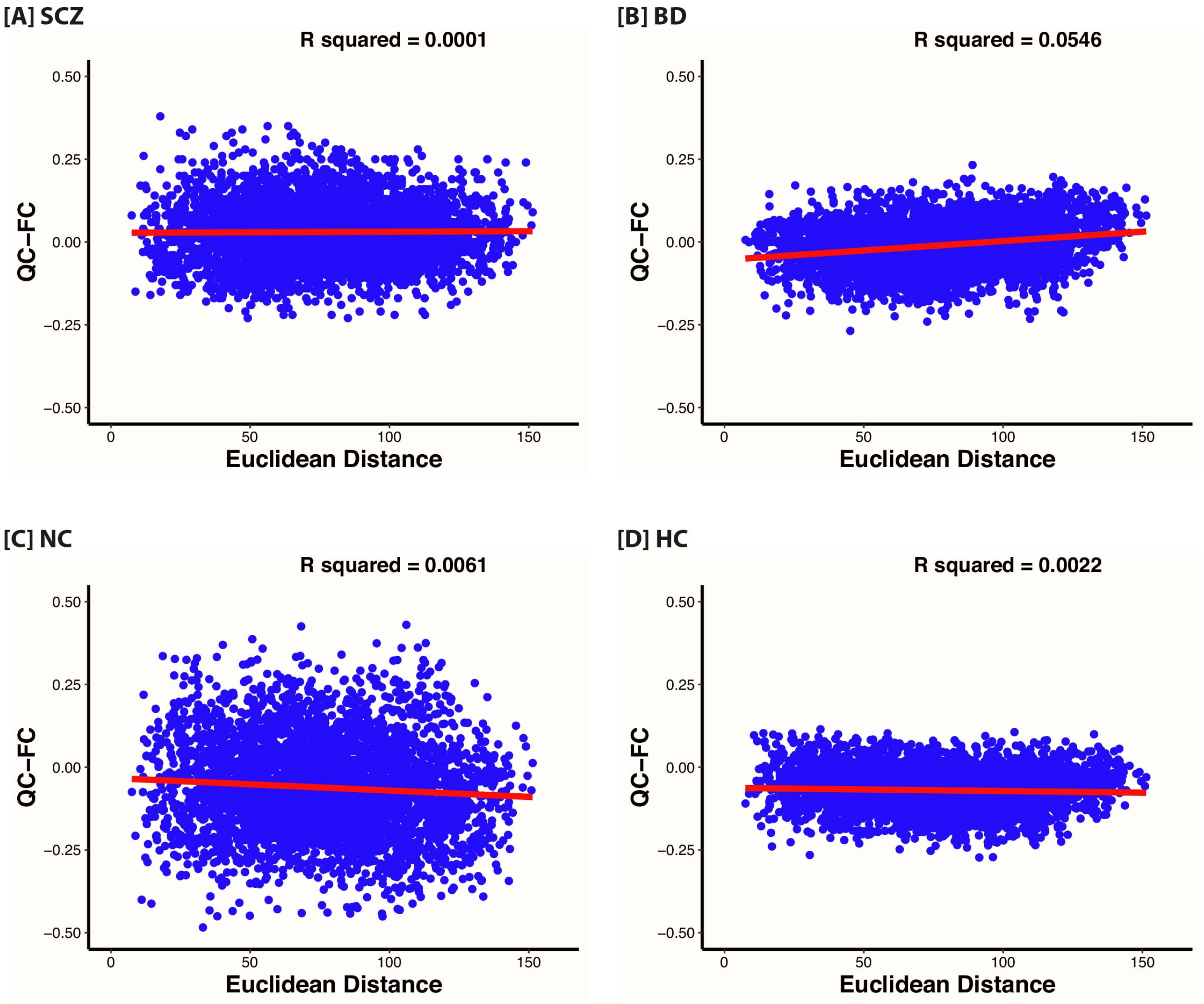

Supplementary Figure 1. Distance dependent effects of motion on edge strength[a].

[a]Euclidean distance each pair of nodes was calculated and compared to the QC-FC (quality control / functional connectivity) correlation. The QC-FC relationship was computed by correlating the network edge weight of the 90-nodes of the AAL atlas with the mean relative RMS (root mean square) motion. The distance dependent effect of motion were minimal in all participant groups. Abbreviations: HC healthy controls; NC non-clinical individuals; BD bipolar disorder; SCZ schizophrenia spectrum disorder.

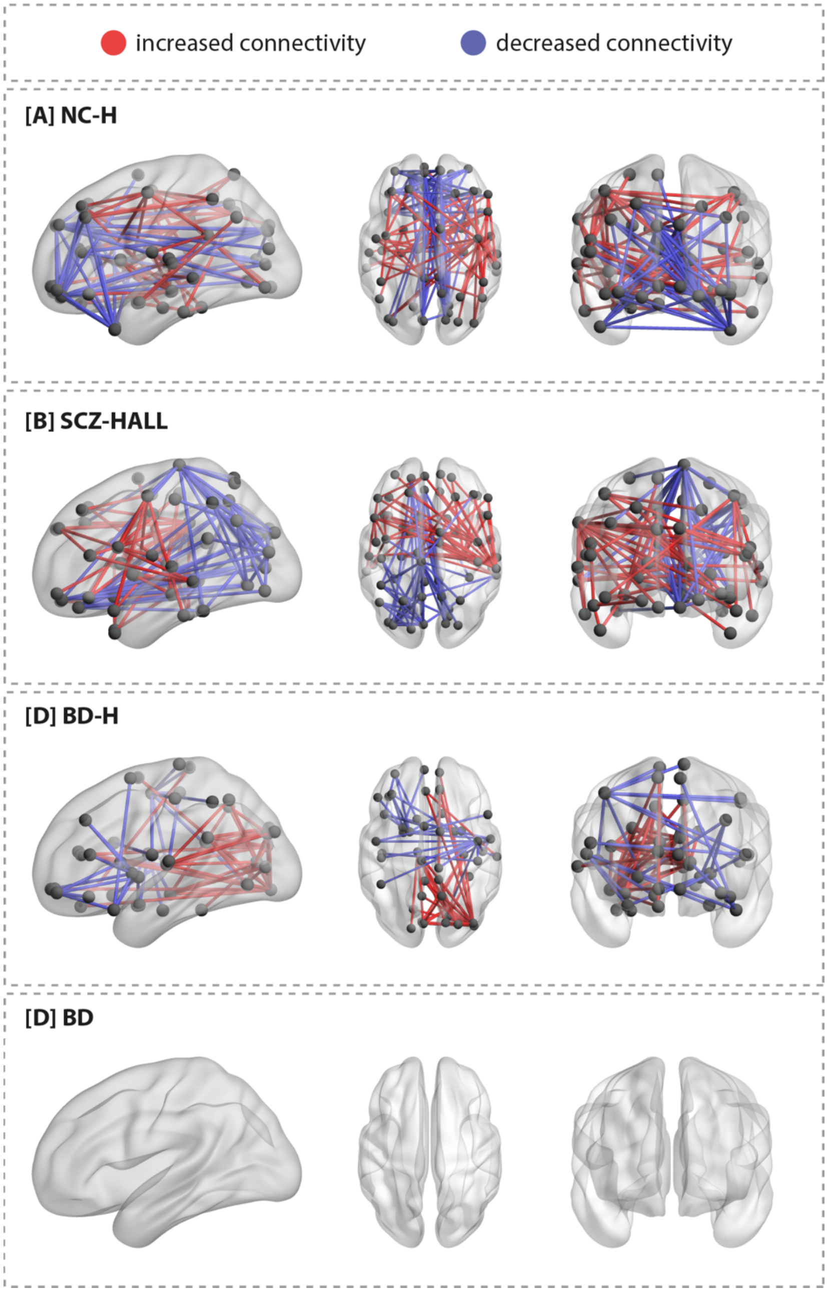

Supplementary Figure 2. Functional connectome alterations in matched subgroups.

Results of the Network Based Statistics between: a) non-clinical individuals with hallucinations (n = 28) vs healthy controls (n = 28); b) schizophrenia patients with hallucinations (n = 45) vs healthy controls (n =45); c) bipolar-I disorder patients with lifetime history of hallucinations (n = 58) vs healthy controls (n= 58). d) bipolar-I disorder patients without a lifetime history of psychosis (n = 32 did not differ from controls (n = 32). The grey dots represent stereotactic centroids of brain regions defined by the AAL atlas. The edges are color-coded based on either an increase (red) or decrease (blue) in connectivity compared to controls. Group differences were tested at a significance level of $P < 0.05$ FWE corrected; the BD-H is tested at a more lenient threshold of $P < 0.10$. Abbreviations: NC-H individuals with non-clinical hallucinations; BD-H bipolar disorder with lifetime history of hallucinations; SCZ-H schizophrenia spectrum disorder with hallucinations.

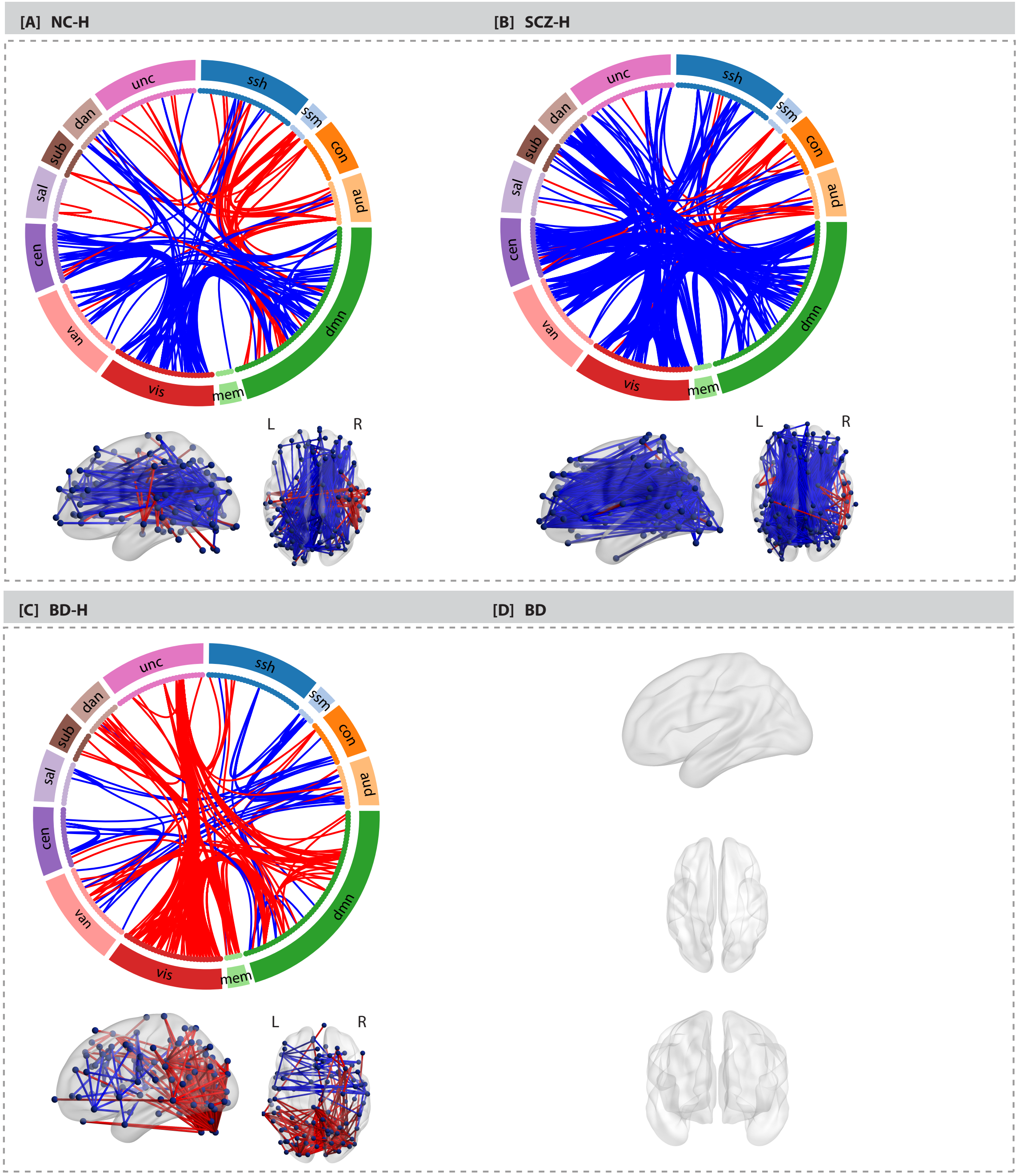

Supplementary Figure 3. Results of the network based statistics analyses using the Power-260 atlas.

Results of the Network Based Statistics analysis between: a) non-clinical individuals with hallucinations (n = 35) vs healthy controls (n = 222); b) schizophrenia patients with hallucinations (n = 95) vs healthy controls (n = 222); c) bipolar-I disorder patients with lifetime history of hallucinations (n = 73) vs healthy controls (n= 222). d) bipolar-I disorder patients without a lifetime history of psychosis (n = 40) did not differ from controls (n = 222). The dark blue dots represent stereotactic centroids of brain regions defined by the Power atlas. The edges are color-coded based on either an increase (red) or decrease (blue) in connectivity compared to controls. Group differences were tested at a significance level of $P < 0.05$ FWE corrected. The corresponding test-statistics, a table with altered connections can be found in the Supplementary Tables 10-12. Abbreviations: AUD auditory network; BD bipolar-I disorder without lifetime history of hallucinations or delusions; BD-H bipolar-I disorder with lifetime history of hallucinations; CEN central-executive network; CER cerebellum; CON cingulo-opercular network; DAN dorsal attention network; DMN default mode network; MEM memory; NC-H individuals with non-clinical hallucinations; SAL salience; SCZ-H schizophrenia spectrum disorder with hallucinations; SSH somatosensory hand; SSM somatosensory mouth; SUB subcortical; VAN ventral attention network; VIS visual.

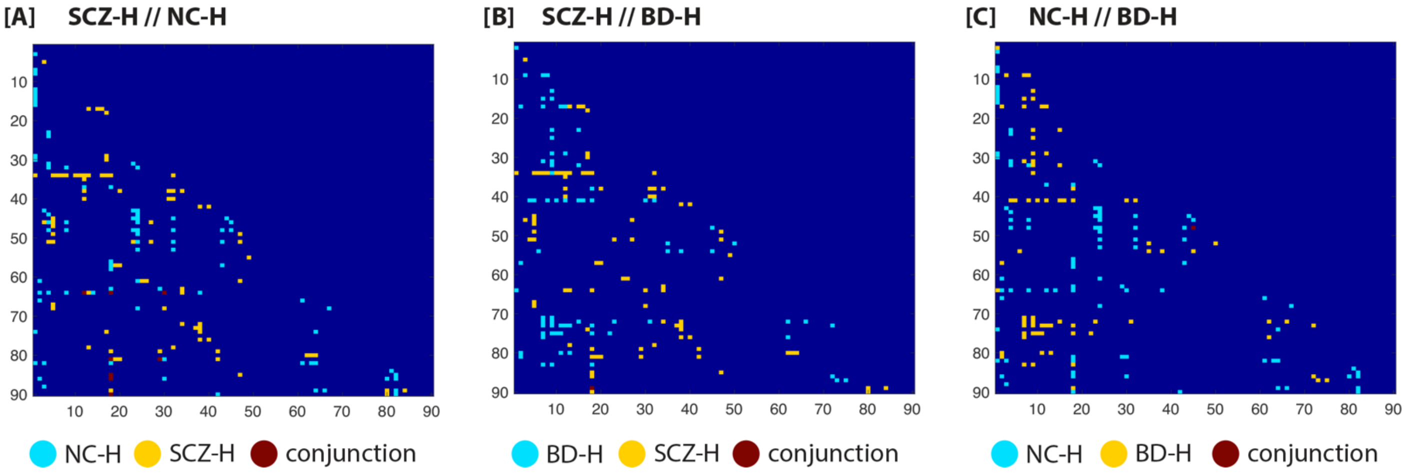

Supplementary Figure 4. Conjunction analysis on the NBS networks of NC-H, SCZ-H and BD-H. a) eight overlapping edges were found between NC-H and SCZ-H. b) One overlapping edge was found between BD-H and SCZ-H, c) one edge for NC-H and BD-H. Results are depicted in matrices of the 90 regions of the AAL atlas. Abbreviations: NC-H individuals with non-clinical hallucinations; BD-H bipolar disorder with hallucinations; SCZ-H schizophrenia spectrum disorder with hallucinations.

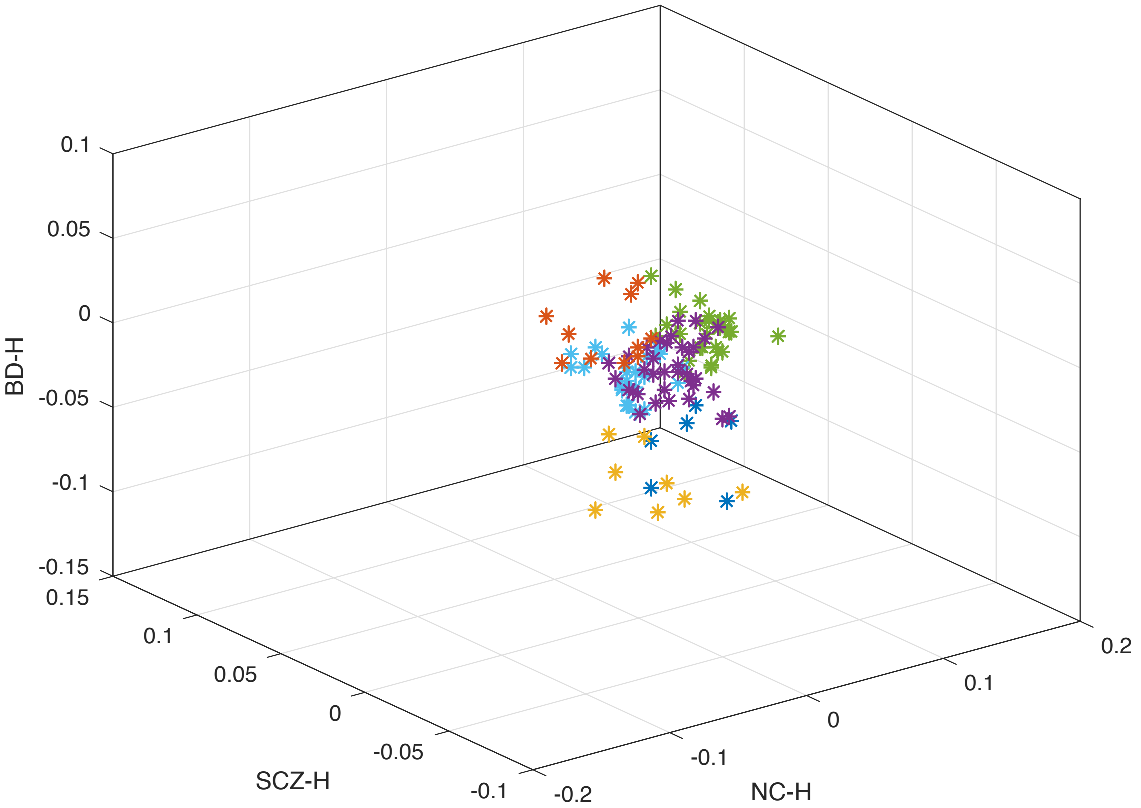

Supplementary Figure 5. Clusters defined by elbow method for k-means clustering.

Six sets of clusters were validated by the elbow method and entered into the k-means clustering algorithm. After a NBS analysis across the three hallucinating groups (NC-H, SCZ-H, BD-H), those edges that were found to significantly differ in functional connectivity across the three groups, were entered into the k-means clustering algorithm. A list of these edges can be found in Supplementary Table 9. Abbreviations: NC-H individuals with non-clinical hallucinations; BD-H bipolar disorder with lifetime history of hallucinations; SCZ-H schizophrenia spectrum disorder with hallucinations.

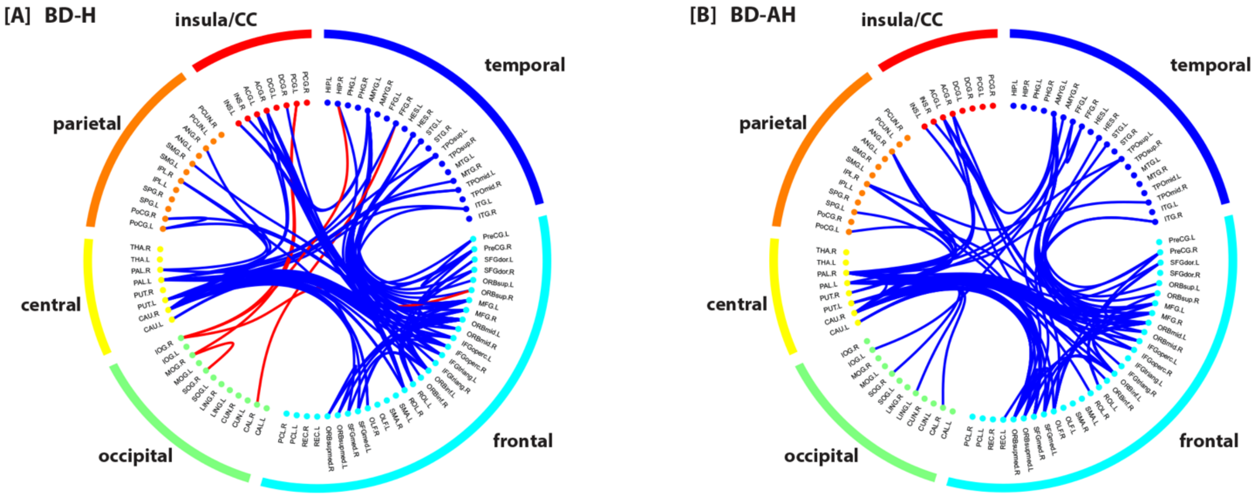

Supplementary Figure 6. Functional connectome disturbances in BD-H and BD-AH. Results of the Network Based Statistics between: a) bipolar-I disorder patients with hallucinations in any modality (n = 74) vs healthy controls (n = 228); b) bipolar-I disorder patients with auditory hallucinations only (n = 39) vs healthy controls (n = 228). The grey dots represent stereotactic centroids of brain regions defined by the AAL atlas. The edges are color-coded based on either an increase (red) or decrease (blue) in connectivity compared to controls. Group differences were tested at a significance level of $P < 0.05$ FWE corrected. The corresponding test-statistics for BD-H is T = 8.5, and for BD-AH T = 9.0. Abbreviations: BD-AH bipolar-I disorder with lifetime history of auditory hallucinations; BD-H bipolar-I disorder with lifetime history of hallucinations.

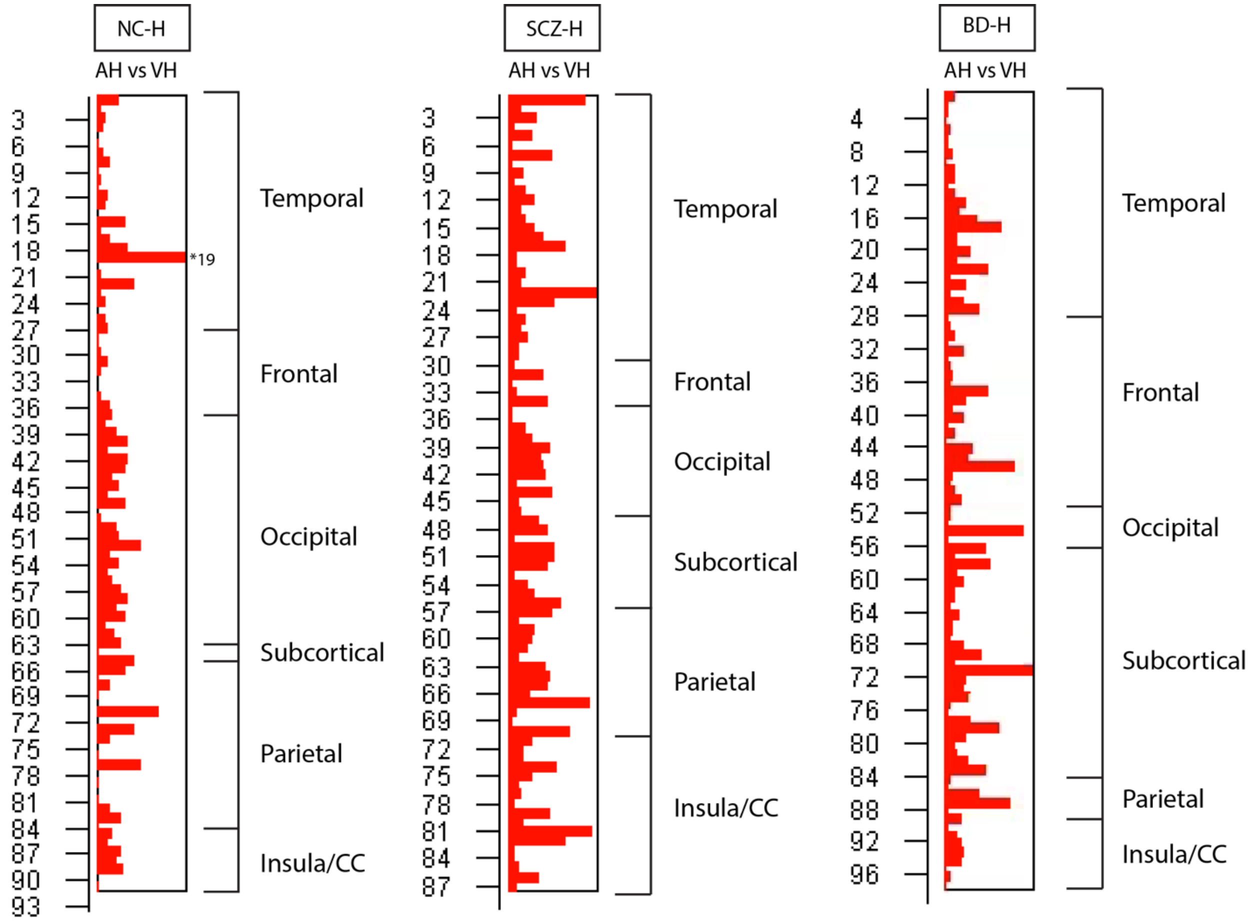

Supplementary Figure 7. Random forest classification based on the connections of the NBS network.

Variable importance scores in the three main classifications. Features concern all connections that are part of the NBS network of each group comparison, see Supplementary Tables 13 – 15 for the included features. The features are ordered according to the lobes of the brain in which the connections originate. VIMP scores showing the relative importance of features for discrimination between visual versus no visual, or visual versus auditory hallucinations. The red bars indicate the variable importance score (VIMP), which ranges on a scale from 0–1. The numbers in front of the red bars indicate the feature number corresponding to each edge pair presented in Supplementary Tables 13 - 15. The most discriminating feature in the NC-H group is indicated with an asterisk (*). Sample sizes are NC-H with only auditory hallucinations n = 7 versus NC-H with auditory and visual hallucinations n = 33; SCZ-H with only auditory hallucinations n = 31 versus SCZ-H with auditory and visual hallucinations n = 53; BD-H with only auditory hallucinations n = 14 versus BD-H with only visual hallucinations n = 25. Number of cases with missing data: 15 visual hallucinations in SCZ-H; 2 auditory hallucinations in BD-H. Abbreviations: AH auditory hallucinations; BD-H bipolar-I disorder with lifetime history of hallucinations; CC cingulate cortex; NC-H individuals with non-clinical hallucinations; SCZ-H schizophrenia spectrum disorder with hallucinations; VH visual hallucinations.

Supplementary Table 6. Results of the network based statistics for non-clinical individuals with hallucinations versus healthy controls using the AAL atlas[a] .

Non-clinical individuals with hallucinations versus healthy controls					
**Region**	**Region**	**Stats[b]**	**Region**	**Region**	**Stats[b]**
Rolandic_Oper_R	Hippocampus_L.	29.48	Cingulum_Ant_R	Occipital_Mid_L.	15.39
Rolandic_Oper_R	Temporal_Mid_R.	26.35	Precentral_R	Parietal_Inf_L.	15.38
Rolandic_Oper_R	SupraMarginal_R.	26.09	Cingulum_Ant_R	Occipital_Inf_L.	15.31
Rolandic_Oper_R	Temporal_Mid_L.	24.91	Rolandic_Oper_R	Postcentral_R.	15.26
Frontal_Sup_Medial_R	Cingulum_Ant_R.	24.42	Temporal_Sup_R	Temporal_Mid_R.	15.25
Frontal_Sup_R	Frontal_Sup_Medial_R.	23.02	Precentral_L	Putamen_R.	15.19
Precentral_L	Insula_L.	22.82	Frontal_Sup_Medial_L	Cingulum_Ant_L.	15.14
Precentral_R	SupraMarginal_R.	22.31	Frontal_Sup_R	Calcarine_R.	14.95
Precentral_L	Frontal_Sup_L.	21.94	Precentral_L	Frontal_Mid_L.	14.73
Precentral_L	Frontal_Inf_Orb_R.	21.22	Amygdala_R	Temporal_Inf_R.	14.72
Rolandic_Oper_R	Temporal_Sup_L.	21.17	Temporal_Sup_R	Temporal_Inf_L.	14.69
Insula_R	Temporal_Sup_L.	20.84	Precentral_L	Temporal_Sup_R.	14.64
Insula_R	SupraMarginal_R.	20.37	Frontal_Sup_Medial_R	Cuneus_R.	14.62
Frontal_Sup_Medial_R	Occipital_Inf_L.	20.21	Precentral_L	Frontal_Inf_Oper_R.	14.60
Calcarine_R	Cuneus_L.	20.16	Frontal_Sup_Medial_L	Calcarine_L.	14.53
Frontal_Sup_R	Frontal_Sup_Medial_L.	20.14	Frontal_Mid_R	Lingual_R.	14.53
Frontal_Sup_R	SupraMarginal_R.	19.71	Cuneus_L	Lingual_R.	14.49
Frontal_Sup_Medial_R	Calcarine_R.	19.51	Cingulum_Ant_R	Cuneus_L.	14.47
Frontal_Inf_Oper_R	Hippocampus_L.	19.29	Precentral_L	Frontal_Inf_Tri_R.	14.44
Rolandic_Oper_R	SupraMarginal_L.	19.13	Frontal_Sup_Medial_R	Occipital_Sup_L.	14.38
Precentral_L	Frontal_Inf_Orb_L.	18.46	Calcarine_L	Postcentral_L.	14.34
Cuneus_L	Cuneus_R.	18.45	Insula_L	SupraMarginal_L.	14.28
Frontal_Sup_L	Temporal_Sup_R.	18.40	Frontal_Inf_Tri_R	SupraMarginal_R.	14.17
Frontal_Sup_Medial_R	Lingual_R.	18.24	Heschl_R	Temporal_Mid_R.	14.10
Precentral_R	Angular_R.	18.17	Cingulum_Ant_R	Lingual_R.	14.06
Frontal_Sup_Medial_R	Occipital_Mid_L.	18.10	Frontal_Sup_Medial_R	Parietal_Inf_L.	14.05
Frontal_Sup_Medial_R	Calcarine_L.	18.08	Angular_R	Temporal_Inf_L.	14.03
Frontal_Mid_R	SupraMarginal_R.	18.02	Frontal_Sup_R	Cuneus_R.	13.98
Frontal_Inf_Oper_R	SupraMarginal_R.	17.71	SupraMarginal_R	Temporal_Inf_L.	13.93
Frontal_Sup_Medial_R	Precuneus_R.	17.52	Calcarine_L	Lingual_R.	13.84
Frontal_Sup_Medial_R	Cuneus_L.	17.49	Frontal_Sup_Medial_L	Lingual_R.	13.71
Frontal_Sup_R	Cingulum_Ant_R.	17.42	Temporal_Sup_R	Temporal_Inf_R.	13.61
Precentral_R	Temporal_Mid_R.	17.24	Precentral_L	Insula_R.	13.60
Frontal_Sup_Medial_L	Cuneus_L.	16.90	Temporal_Sup_L	Temporal_Pole_Sup_R.	13.57
SupraMarginal_L	Temporal_Sup_R.	16.61	Frontal_Sup_L	Calcarine_L.	13.46
Rolandic_Oper_R	Postcentral_L.	16.57	Parietal_Inf_L	Angular_R.	13.42
Rolandic_Oper_R	Temporal_Sup_R.	16.42	Frontal_Sup_R	Cingulum_Ant_L.	13.35
SupraMarginal_R	Temporal_Sup_R.	16.42	Insula_L	Temporal_Sup_L.	13.30
SupraMarginal_R	Putamen_R.	16.06	Rolandic_Oper_R	Fusiform_R.	13.25
Insula_R	Temporal_Mid_R.	15.88	Frontal_Mid_R	Cuneus_R.	13.21
Precentral_L	Frontal_Inf_Tri_L.	15.87	Parietal_Inf_L	Temporal_Sup_R.	13.13
Hippocampus_R	SupraMarginal_R.	15.87	Precentral_L	Frontal_Mid_R.	13.12
Precuneus_L	Precuneus_R.	15.74	Frontal_Mid_R	Cingulum_Ant_R.	13.11
Rolandic_Oper_R	Temporal_Inf_R.	15.63	Frontal_Sup_L	Temporal_Pole_Mid_R.	13.03
Temporal_Sup_R	Temporal_Mid_L.	15.54	Calcarine_L	Occipital_Mid_L.	13.00
Frontal_Sup_R	Lingual_R.	15.51			

[a]NBS F-test at T = 13.0; $P < 0.05$ with 10,000 permutations, adjusted for multiple comparisons. [b]Stats indicates the T-statistic of significant edges.

Supplementary Table 7. Results of the network based statistics for schizophrenia patients with hallucinations versus healthy controls using the AAL atlas[a] .

Schizophrenia patients with hallucinations versus healthy controls					
**Region**	**Region**	**Stats[b]**	**Region**	**Region**	**Stats[b]**
Hippocampus_R	Pallidum_R.	25.06	Frontal_Inf_Oper_R	Hippocampus_R.	14.98
Rolandic_Oper_R	Temporal_Mid_R.	24.22	Frontal_Sup_Orb_R	Cingulum_Mid_R.	14.95
ParaHippocampal_R	Amygdala_R.	23.42	Rolandic_Oper_L	Rolandic_Oper_R.	14.84
Rolandic_Oper_R	SupraMarginal_R.	23.26	Frontal_Sup_Orb_L	Occipital_Mid_L.	14.69
Rolandic_Oper_L	Insula_L.	21.78	Frontal_Inf_Oper_R	Cingulum_Post_L.	14.58
Rolandic_Oper_R	Temporal_Inf_R.	20.97	Insula_L	Temporal_Sup_L.	14.37
Lingual_L	Occipital_Mid_L.	20.96	Supp_Motor_Area_R	Temporal_Sup_L.	14.20
Frontal_Inf_Oper_R	Cingulum_Mid_R.	20.71	Rectus_L	Cuneus_R.	14.10
Frontal_Mid_L	Cingulum_Mid_R.	20.06	Rolandic_Oper_L	Putamen_R.	14.09
Cingulum_Mid_R	Hippocampus_R.	19.93	Rolandic_Oper_R	Temporal_Mid_L.	14.08
Cingulum_Ant_L	ParaHippocampal_R.	19.86	Insula_R	Precuneus_R.	14.04
Insula_L	Heschl_L.	19.38	Frontal_Inf_Tri_L	Rolandic_Oper_L.	14.01
Rolandic_Oper_R	Cingulum_Mid_R.	19.32	Frontal_Inf_Tri_L	SupraMarginal_R.	13.99
Hippocampus_R	Putamen_R.	19.28	Frontal_Mid_R	Cingulum_Mid_R.	13.97
Frontal_Inf_Oper_L	Cingulum_Mid_R.	19.14	Frontal_Sup_Orb_L	Precuneus_R.	13.96
Supp_Motor_Area_R	Postcentral_L.	18.57	Frontal_Med_Orb_R	Parietal_Inf_L.	13.95
SupraMarginal_L	Heschl_R.	18.32	Frontal_Sup_Medial_L	Occipital_Mid_L.	13.94
Rolandic_Oper_L	Insula_R.	18.21	Heschl_R	Temporal_Inf_R.	13.94
Rolandic_Oper_L	Cingulum_Mid_R.	17.75	Cingulum_Ant_R	Cingulum_Mid_R.	13.93
Frontal_Inf_Tri_L	Cingulum_Mid_R.	17.40	Supp_Motor_Area_R	Hippocampus_R.	13.87
Cingulum_Mid_R	SupraMarginal_L.	17.05	Frontal_Sup_Orb_L	Lingual_L.	13.82
Frontal_Mid_Orb_R	Cingulum_Mid_R.	17.03	Lingual_L	Occipital_Sup_L.	13.81
Cingulum_Ant_R	Thalamus_R.	16.88	Heschl_R	Temporal_Inf_L.	13.80
Supp_Motor_Area_L	Temporal_Sup_L.	16.82	ParaHippocampal_R	Pallidum_R.	13.78
Frontal_Sup_L	Frontal_Sup_Orb_L.	16.79	Frontal_Sup_L	Cuneus_R.	13.73
Frontal_Inf_Tri_L	Thalamus_R.	16.79	Lingual_L	Parietal_Inf_L.	13.55
Frontal_Sup_Orb_L	Precuneus_L.	16.71	Occipital_Sup_L	Fusiform_L.	13.53
Frontal_Sup_Orb_L	Cingulum_Mid_R.	16.69	Amygdala_R	Heschl_L.	13.48
Cingulum_Ant_R	ParaHippocampal_R.	16.40	Temporal_Pole_Sup_R	Temporal_Inf_L.	13.44
Supp_Motor_Area_L	Postcentral_L.	16.28	Frontal_Med_Orb_L	Parietal_Inf_L.	13.40
Frontal_Inf_Orb_R	Rolandic_Oper_L.	16.19	Frontal_Inf_Orb_R	Cingulum_Mid_R.	13.39
Hippocampus_L	Putamen_L.	16.11	Frontal_Inf_Oper_R	ParaHippocampal_R.	13.33
Hippocampus_R	Amygdala_R.	16.10	Cingulum_Mid_R	Caudate_R.	13.32
Cingulum_Ant_R	Hippocampus_R.	15.79	SupraMarginal_R	Heschl_R.	13.32
Rolandic_Oper_R	Heschl_L.	15.69	Rectus_L	Occipital_Mid_L.	13.28
Parietal_Inf_R	Heschl_R.	15.67	Insula_R	SupraMarginal_R.	13.21
Cingulum_Ant_L	Hippocampus_R.	15.51	Hippocampus_R	Caudate_R.	13.21
Frontal_Sup_R	Occipital_Mid_L.	15.44	Amygdala_R	Temporal_Sup_L.	13.21
Frontal_Sup_Orb_L	Cuneus_L.	15.41	Rolandic_Oper_R	Temporal_Inf_L.	13.17
Frontal_Inf_Orb_L	Rolandic_Oper_L.	15.38	Frontal_Sup_Orb_L	Cuneus_R.	13.12
Frontal_Sup_Orb_L	Occipital_Sup_L.	15.34	Frontal_Inf_Oper_R	SupraMarginal_R.	13.12
Rolandic_Oper_R	Temporal_Sup_L.	15.30	Cingulum_Mid_R	SupraMarginal_R.	13.12
Precentral_L	Cingulum_Mid_R.	15.27	Lingual_L	Temporal_Mid_L.	13.01
Hippocampus_R	Putamen_L.	15.06			

[a]NBS F-test at T = 13.0; $P < 0.05$ with 10,000 permutations, adjusted for multiple comparisons. [b]Stats indicates the T-statistic of significant edges.

Supplementary Table 8. Results of the network based statistics for bipolar-I disorder patients with hallucinations versus healthy controls using the AAL atlas[a].

Bipolar-I disorder patients with hallucinations versus healthy controls					
**Region**	**Region**	**Stats[b]**	**Region**	**Region**	**Stats[b]**
Frontal Sup L	Frontal Mid Orb L.	25.31	Frontal Mid L	Caudate L.	10.11
Precentral R	Heschl R.	24.24	Frontal Mid L	Frontal Inf Orb L.	10.06
Frontal Mid Orb L	Frontal Inf Orb L.	22.95	Frontal Mid Orb R	Pallidum L.	10.02
Precentral R	Postcentral L.	21.81	Frontal Sup Orb R	Occipital Inf R.	10.00
Frontal Mid Orb L	Frontal Sup Medial L.	19.02	Hippocampus R	Occipital Inf R.	10.00
Frontal Mid Orb L	Pallidum L.	16.99	Putamen L	Temporal Pole Mid L.	10.00
Frontal Mid L	Frontal Mid Orb L.	16.25	Frontal Inf Orb L	Amygdala L.	9.93
Frontal Mid R	Frontal Mid Orb L.	16.24	Frontal Mid L	Putamen L.	9.93
Frontal Mid Orb L	Temporal Pole Sup L.	16.13	Frontal Mid Orb L	Cingulum Ant R.	9.90
Frontal Mid Orb L	Cingulum Ant L.	15.41	Rolandic Oper R	Putamen L.	9.88
Frontal Mid Orb L	Frontal Med Orb L.	15.35	Frontal Mid L	Rolandic Oper L.	9.86
Precentral R	Rolandic Oper L.	15.10	Caudate R	Putamen L.	9.69
Frontal Inf Oper L	Putamen L.	15.07	Rolandic Oper R	Hippocampus R.	9.67
Precentral L	Precentral R.	14.52	Frontal Mid R	Frontal Inf Orb L.	9.66
Frontal Mid Orb L	Frontal Inf Tri L.	14.22	Precentral R	Postcentral R.	9.57
Frontal Sup Medial L	Caudate R.	13.31	Rolandic Oper R	Temporal Pole Sup L.	9.57
Cingulum Ant L	Caudate R.	12.80	Rolandic Oper R	Temporal Inf L.	9.56
Frontal Inf Oper R	Putamen L.	12.50	Frontal Mid L	Pallidum R.	9.53
Occipital Sup R	Occipital Mid R.	12.49	Cingulum Post L	Occipital Mid R.	9.43
Frontal Inf Oper R	Amygdala L.	12.39	Frontal Mid R	Rolandic Oper L.	9.37
Cingulum Ant R	Amygdala L.	12.36	Frontal Mid L	Cingulum Ant L.	9.36
Frontal Inf Oper L	Pallidum L.	12.36	Frontal Inf Orb L	Cingulum Ant L.	9.34
Frontal Mid R	Amygdala L.	12.30	Frontal Inf Oper R	Rolandic Oper L.	9.28
Rolandic Oper R	Amygdala L.	12.26	Caudate R	Temporal Mid R.	9.25
Frontal Sup Orb L	Amygdala L.	12.25	Frontal Mid L	Frontal Med Orb R.	9.24
Frontal Inf Tri L	Putamen L.	12.04	Frontal Inf Orb L	Frontal Sup Medial R.	9.20
Frontal Mid L	Caudate R.	11.98	Frontal Mid Orb R	Amygdala L.	9.20
Rolandic Oper R	Pallidum L.	11.69	Frontal Mid Orb L	Cingulum Mid R.	9.14
Pallidum L	Temporal Pole Mid L.	11.68	Olfactory R	Putamen L.	9.13
Frontal Inf Oper L	Rolandic Oper L.	11.46	Frontal Med Orb L	Frontal Med Orb R.	9.08
Frontal Mid Orb L	Caudate L.	11.44	Frontal Inf Tri R	Amygdala L.	8.98
Frontal Inf Tri L	Heschl R.	11.44	Calcarine R	Fusiform R.	8.98
Precentral R	Temporal Sup L.	11.30	Fusiform R	Caudate R.	8.94
Angular R	Caudate R.	11.28	Frontal Mid Orb L	Putamen L.	8.94
Parietal Inf R	Pallidum R.	11.27	Frontal Mid Orb L	Frontal Med Orb R.	8.93
Parietal Inf R	Caudate R.	11.09	Frontal Mid L	Pallidum L.	8.91
Frontal Mid L	Temporal Pole Sup L.	10.83	Cingulum Ant R	Caudate R.	8.81
Frontal Mid Orb L	Caudate R.	10.79	Pallidum L	Temporal Pole Sup L.	8.81
Frontal Mid Orb L	Insula L.	10.78	Frontal Mid L	Amygdala L.	8.79
Frontal Inf Orb L	Frontal Sup Medial L.	10.76	Frontal Sup Medial R	Parietal Inf R.	8.74
Olfactory R	Pallidum L.	10.66	Frontal Mid Orb L	Frontal Inf Oper R.	8.70
Frontal Sup R	Amygdala L.	10.58	Frontal Sup Orb R	Amygdala L.	8.62
Frontal Inf Oper L	Heschl R.	10.57	Frontal Sup R	Frontal Mid Orb L.	8.58
Precentral L	SupraMarginal R.	10.53	Frontal Sup L	Frontal Inf Orb L.	8.58
Frontal Inf Orb L	Cingulum Ant R.	10.43	Cingulum Post L	Occipital Inf R.	8.58
Frontal Inf Orb R	Amygdala L.	10.31	Frontal Inf Oper R	Pallidum L.	8.58
Insula R	Amygdala L.	10.21	Postcentral L	Postcentral R.	8.51
Frontal Inf Oper R	Insula L.	10.18	Caudate R	Temporal Inf L.	8.51
Frontal Inf Orb L	Caudate R.	10.13			

[a]NBS F-test at T = 8.5; $P < 0.05$ with 10,000 permutations, adjusted for multiple comparisons. [b]Stats indicates the T-statistic of significant edges.

Supplementary Table 9. Results of the network based statistics across all three hallucination groups using the AAL atlas.

Comparison across all three hallucination groups					
Region	Region	Cluster	Region	Region	Cluster
Precentral R	Precentral L	1	Temporal Pole Sup	SupraMarginal L	4
Postcentral L	Precentral R	1	Thalamus R	Pallidum L	4
Postcentral R	Precentral R	1	Temporal Mid R	Temporal Pole Sup	4
Rolandic Oper L	Frontal Inf Oper L	1	Frontal Sup L	Precentral L	5
Hippocampus R	Rolandic Oper R	1	Frontal Inf Oper R	Precentral L	5
Heschl R	Rolandic Oper R	1	Frontal Inf Tri L	Precentral L	5
Lingual R	Calcarine L	2	Frontal Inf Orb L	Precentral L	5
Occipital Mid L	Calcarine L	2	Frontal Inf Orb R	Precentral L	5
Cuneus L	Calcarine R	2	SupraMarginal R	Frontal Sup R	5
Fusiform R	Calcarine R	2	SupraMarginal R	Frontal Mid R	5
Lingual R	Cuneus L	2	SupraMarginal R	Frontal Inf Oper R	5
Occipital Inf R	Cuneus L	2	Temporal Mid R	Frontal Inf Oper R	5
Precuneus R	Cuneus L	2	Hippocampus L	Rolandic Oper R	5
Fusiform R	Cuneus R	2	Fusiform R	Rolandic Oper R	5
Occipital Mid L	Lingual L	2	Postcentral L	Rolandic Oper R	5
Occipital Mid L	Occipital Sup L	2	Postcentral R	Rolandic Oper R	5
Precuneus L	Occipital Inf R	2	SupraMarginal L	Rolandic Oper R	5
Cingulum Mid R	Frontal Mid Orb L	3	SupraMarginal R	Rolandic Oper R	5
Putamen L	Frontal Inf Oper L	3	Temporal Sup L	Rolandic Oper R	5
Pallidum L	Frontal Inf Oper L	3	Temporal Sup R	Rolandic Oper R	5
Heschl R	Frontal Inf Oper L	3	Temporal Mid L	Rolandic Oper R	5
Putamen L	Frontal Inf Oper R	3	Temporal Mid R	Rolandic Oper R	5
Pallidum L	Frontal Inf Oper R	3	Temporal Inf R	Rolandic Oper R	5
Putamen R	Putamen L	3	SupraMarginal L	Insula L	5
Pallidum L	Putamen R	3	SupraMarginal L	Insula R	5
Rolandic Oper L	Frontal Sup L	4	SupraMarginal R	Insula R	5
Heschl R	Frontal Sup L	4	Temporal Sup L	Insula R	5
Temporal Inf L	Frontal Sup L	4	Temporal Sup R	Insula R	5
Rolandic Oper L	Frontal Sup R	4	Temporal Mid R	Insula R	5
Thalamus R	Frontal Sup R	4	SupraMarginal R	Hippocampus R	5
Heschl R	Frontal Mid L	4	Temporal Mid L	Heschl R	5
Temporal Sup L	Frontal Mid L	4	Temporal Pole Sup	Temporal Sup L	5
Rolandic Oper L	Frontal Mid R	4	SupraMarginal R	Precentral L	6
Rolandic Oper L	Frontal Mid Orb L	4	Thalamus R	Frontal Sup Orb L	6
Hippocampus L	Frontal Inf Oper R	4	Rolandic Oper L	Frontal Mid L	6
SupraMarginal L	Frontal Inf Oper R	4	Cingulum Mid R	Frontal Mid L	6
Heschl R	Frontal Inf Oper R	4	SupraMarginal L	Frontal Mid L	6
Temporal Sup L	Frontal Inf Oper R	4	SupraMarginal R	Frontal Mid L	6
Heschl R	Frontal Inf Tri L	4	Temporal Inf R	Frontal Mid R	6
Temporal Sup L	Frontal Inf Tri L	4	Temporal Sup L	Frontal Inf Oper L	6
Rolandic Oper L	Frontal Inf Tri R	4	Rolandic Oper L	Frontal Inf Oper R	6
Cingulum Mid R	Frontal Inf Orb L	4	Cingulum Mid R	Frontal Inf Oper R	6
Hippocampus L	Frontal Inf Orb L	4	Hippocampus R	Frontal Inf Oper R	6
Thalamus R	Frontal Inf Orb L	4	Thalamus R	Frontal Inf Oper R	6
Temporal Sup L	Frontal Inf Orb L	4	Rolandic Oper L	Frontal Inf Tri L	6
Hippocampus L	Frontal Inf Orb R	4	Rolandic Oper L	Frontal Inf Orb L	6
Thalamus R	Frontal Inf Orb R	4	Rolandic Oper L	Frontal Inf Orb R	6
Heschl R	Frontal Inf Orb R	4	Rolandic Oper R	Rolandic Oper L	6
Temporal Sup L	Frontal Inf Orb R	4	Insula R	Rolandic Oper L	6
Occipital Mid R	Rolandic Oper R	4	Cingulum Mid R	Rolandic Oper R	6
Caudate L	Rolandic Oper R	4	Parietal Inf R	Rolandic Oper R	6
Parietal Inf R	Insula L	4	Temporal Inf L	Rolandic Oper R	6
Hippocampus L	Insula R	4	Hippocampus R	Insula R	6
Postcentral R	Insula R	4	Thalamus R	Insula R	6
Putamen L	Hippocampus L	4	Heschl R	Insula R	6
Temporal Pole Sup	Hippocampus L	4	Heschl R	SupraMarginal R	6
Heschl R	Occipital Mid R	4	Thalamus R	Putamen R	6

Supplementary Table 10. Results of the network based statistics for non-clinical individuals with hallucinations versus healthy controls using the Power atlas[a].

Non-clinical individuals with hallucinations versus healthy controls										
RSN	RSN	Stats		RSN	RSN	Stats		RSN	RSN	Stats
aud	dmn	33.95		vis	vis	15.57		vis	cen	13.85
dmn	dmn	27.84		dmn	vis	15.53		dmn	dmn	13.84
dmn	dmn	26.79		ssm	van	15.50		vis	vis	13.84
ssh	dmn	26.54		dmn	dan	15.48		vis	vis	13.84
ssm	dmn	25.98		vis	vis	15.45		vis	sal	13.84
unc	ssm	25.22		aud	vis	15.43		vis	vis	13.83
aud	dmn	25.13		vis	vis	15.39		aud	dmn	13.82
vis	vis	24.51		vis	vis	15.34		dmn	sal	13.82
vis	cen	24.32		aud	unc	15.28		dmn	vis	13.81
aud	dmn	21.16		dmn	dmn	15.26		dmn	vis	13.77
aud	dmn	21.11		dmn	vis	15.26		dmn	cen	13.76
ssm	con	21.07		vis	vis	15.25		dmn	vis	13.74
vis	vis	20.87		ssm	aud	15.22		sal	van	13.73
dmn	vis	20.65		dmn	sal	15.17		dmn	vis	13.72
ssh	dmn	20.41		vis	dan	15.17		ssh	vis	13.71
ssh	dmn	20.05		aud	cen	15.13		vis	sal	13.70
dmn	dmn	19.88		vis	sal	15.13		dmn	vis	13.69
dmn	vis	19.85		ssh	dmn	15.02		vis	sal	13.69
con	dmn	19.84		dmn	cen	15.01		dmn	vis	13.67
vis	cen	19.78		aud	dmn	15.00		ssh	vis	13.66
dmn	vis	19.75		dmn	vis	15.00		dmn	dmn	13.65
vis	sal	19.62		ssh	aud	14.94		ssm	dmn	13.64
ssh	van	19.59		vis	cen	14.94		dmn	vis	13.64
vis	vis	19.37		vis	unc	14.93		dmn	dan	13.64
dmn	dmn	19.36		vis	vis	14.90		dmn	vis	13.63
vis	sal	19.23		vis	cen	14.90		dmn	vis	13.62
ssh	dmn	19.07		dmn	cen	14.85		cen	sal	13.60
aud	vis	19.03		dmn	vis	14.83		dmn	dmn	13.57
ssm	aud	18.99		dmn	vis	14.81		dmn	sal	13.57
vis	vis	18.94		dmn	vis	14.80		dmn	unc	13.53
vis	vis	18.77		dmn	vis	14.79		vis	cen	13.53
dmn	vis	18.69		vis	vis	14.77		vis	sal	13.52
dmn	vis	18.55		dmn	vis	14.76		sal	dan	13.52
unc	aud	18.54		ssm	aud	14.75		vis	sal	13.50
vis	vis	18.41		ssh	unc	14.73		vis	sal	13.50
vis	vis	18.21		dmn	vis	14.72		vis	cen	13.47
vis	cen	18.13		vis	cen	14.72		vis	dan	13.46
ssm	dmn	18.03		cen	sal	14.71		dmn	vis	13.45
ssm	aud	18.01		ssm	van	14.71		aud	unc	13.41
cen	sal	17.84		vis	sal	14.70		dmn	vis	13.39
vis	sal	17.83		dmn	vis	14.66		vis	sal	13.39
vis	cen	17.82		sal	dan	14.64		dmn	vis	13.37
vis	sal	17.72		ssh	dmn	14.62		vis	dan	13.36
ssh	aud	17.49		ssh	vis	14.62		dmn	vis	13.35
dmn	vis	17.46		ssh	dmn	14.58		dmn	dan	13.35
cen	sal	17.44		dmn	sub	14.57		dmn	vis	13.32
dmn	vis	17.26		vis	cen	14.56		dmn	vis	13.32
dmn	vis	17.21		aud	dan	14.54		sub	sub	13.32

unc	ssm	17.20		ssm	dmn	14.52		vis	sal	13.29
ssh	dmn	17.12		dmn	vis	14.50		vis	sal	13.27
dmn	vis	17.04		vis	sal	14.47		dmn	vis	13.25
dmn	cen	17.00		dmn	vis	14.44		dmn	sal	13.25
aud	dmn	16.75		vis	vis	14.41		dmn	dan	13.24
aud	aud	16.73		dmn	vis	14.39		ssh	cen	13.22
vis	vis	16.66		sal	sal	14.37		cen	sal	13.22
ssh	dmn	16.55		dmn	dmn	14.36		dmn	dmn	13.21
dmn	vis	16.51		dmn	vis	14.32		dmn	cen	13.21
vis	vis	16.51		con	unc	14.30		dmn	vis	13.20
ssh	ssm	16.49		vis	cen	14.30		vis	vis	13.20
ssh	aud	16.45		dmn	dmn	14.25		con	dmn	13.19
dmn	dmn	16.44		vis	cen	14.25		ssm	dmn	13.18
aud	vis	16.41		dmn	vis	14.21		vis	sal	13.16
dmn	vis	16.34		vis	sal	14.18		dmn	vis	13.14
dmn	sub	16.33		vis	cen	14.17		ssh	dmn	13.13
dmn	mem	16.22		dmn	dmn	14.09		dmn	cen	13.11
vis	sal	16.20		vis	van	14.09		ssh	dmn	13.09
ssh	dmn	16.17		dmn	vis	14.08		dmn	vis	13.08
ssh	sal	16.16		vis	cen	14.01		vis	sal	13.08
ssm	dmn	16.12		dmn	vis	14.00		dmn	dan	13.08
dmn	vis	16.09		vis	dan	14.00		dmn	vis	13.07
vis	cen	15.96		ssh	vis	13.99		vis	sal	13.07
ssm	dmn	15.94		vis	vis	13.98		vis	sal	13.06
dmn	dmn	15.89		vis	cen	13.98		dmn	vis	13.05
vis	dan	15.82		vis	vis	13.97		cen	sal	13.05
vis	sal	15.81		cen	sal	13.96		ssh	ssm	13.04
dmn	unc	15.80		ssm	dmn	13.95		vis	vis	13.04
dmn	dmn	15.74		dmn	vis	13.91		aud	dmn	13.03
vis	sal	15.71		dmn	sal	13.89		ssm	dmn	13.02
dmn	sal	15.70		aud	dmn	13.88				

[a]NBS F-test at T = 13.0; $P < 0.05$ with 10,000 permutations, adjusted for multiple comparisons. [b]Stats indicates the T-statistic of significant edges. Abbreviations; AUD auditory network; CEN central-executive network; CER cerebellum; CON cingulo-opercular network; DAN dorsal attention network; DMN default mode network; MEM memory; RSN Resting State Network; SAL salience; SSH somatosensory hand; SSM somatosensory mouth; SUB subcortical; VAN ventral attention network; VIS visual.

Supplementary Table 11. Results of the network based statistics for schizophrenia patients with hallucinations versus healthy controls using the Power atlas[a] .

Schizophrenia patients with hallucinations versus healthy controls										
**RSN**	**RSN**	**Stats**		**RSN**	**RSN**	**Stats**		**RSN**	**RSN**	**Stats**
vis	vis	34.78		vis	vis	16.01		ssh	dmn	14.24
dmn	cen	29.83		mem	unc	16.01		dmn	vis	14.24
dmn	cen	26.80		vis	sal	16.01		vis	cen	14.24
con	aud	25.70		sal	sal	16.00		vis	cen	14.23
sal	unc	24.79		vis	cen	15.92		ssm	aud	14.22
vis	cen	24.25		sal	dan	15.90		vis	vis	14.21
dmn	dmn	23.91		dmn	vis	15.87		vis	sal	14.21
unc	cen	23.84		dmn	vis	15.87		vis	sal	14.20
vis	vis	23.77		dmn	cen	15.87		dmn	sal	14.20
cen	cen	23.74		cen	sal	15.86		ssh	cen	14.16
aud	sub	23.65		ssh	cen	15.85		dmn	vis	14.14
aud	dmn	23.44		cen	sal	15.85		vis	sal	14.13
vis	sal	23.06		vis	dan	15.84		dmn	vis	14.11
cen	dan	22.76		cen	dan	15.80		dmn	dmn	14.10
dmn	cen	22.72		vis	cen	15.77		vis	van	14.09
ssh	cen	22.11		cen	dan	15.75		sal	dan	14.09
dmn	cen	21.87		ssh	con	15.74		dmn	dmn	14.06
ssh	sal	21.83		vis	sal	15.74		vis	cen	14.05
dmn	sal	21.80		vis	vis	15.72		cen	cen	14.05
vis	cen	21.73		dmn	vis	15.72		dmn	van	14.05
vis	sal	21.33		con	sal	15.72		vis	dan	14.04
cen	dan	21.21		cen	cen	15.70		dmn	cen	13.99
dmn	vis	21.20		ssh	cen	15.70		vis	sal	13.99
dmn	sal	21.19		vis	vis	15.69		ssh	vis	13.97
dmn	vis	20.95		vis	vis	15.68		vis	cen	13.97
ssh	cen	20.75		vis	cen	15.66		vis	sal	13.97
vis	vis	20.73		unc	dan	15.66		vis	vis	13.96
vis	cen	20.55		sal	dan	15.66		ssh	sal	13.95
vis	sal	20.53		dmn	vis	15.64		dmn	vis	13.94
vis	sal	20.52		vis	cen	15.64		dmn	vis	13.94
vis	cen	20.49		dmn	vis	15.61		vis	unc	13.94
dmn	vis	20.39		sal	sal	15.60		dmn	vis	13.93
vis	sal	20.35		ssh	dmn	15.58		vis	sal	13.93
vis	cen	20.11		vis	vis	15.57		mem	sal	13.93
dmn	sal	20.02		dmn	sal	15.55		dmn	vis	13.92
dmn	sal	20.01		vis	unc	15.53		dmn	sal	13.91
vis	vis	19.94		dmn	dan	15.51		cen	cen	13.90
dmn	vis	19.93		dmn	cen	15.49		dmn	cen	13.89
vis	unc	19.92		cen	sal	15.49		dmn	mem	13.88
dmn	sal	19.82		con	dmn	15.47		vis	vis	13.87
vis	sal	19.61		dmn	vis	15.47		vis	cen	13.86
con	aud	19.55		dmn	sal	15.46		vis	vis	13.85
cen	sal	19.54		ssh	cen	15.44		aud	dmn	13.84
dmn	sal	19.52		vis	cen	15.43		dmn	mem	13.83
dmn	dan	19.50		vis	sal	15.43		dmn	dan	13.83
dmn	vis	19.49		dmn	dmn	15.41		dmn	vis	13.82
vis	cen	19.43		dmn	sal	15.38		vis	unc	13.82

dmn	dan	19.42		aud	sal	15.38		sal	dan	13.82
sal	sal	19.26		cen	sal	15.38		vis	cen	13.81
vis	vis	19.25		con	cen	15.34		con	sal	13.81
dmn	sal	19.21		dmn	vis	15.30		dmn	dmn	13.80
vis	sal	19.15		dmn	dmn	15.28		dmn	cen	13.80
aud	cen	19.14		vis	vis	15.28		mem	vis	13.79
vis	vis	19.03		vis	van	15.28		sal	dan	13.79
sal	dan	18.96		dmn	vis	15.26		vis	vis	13.78
aud	sal	18.93		unc	aud	15.24		sal	dan	13.77
vis	vis	18.92		dmn	vis	15.24		aud	sal	13.75
vis	vis	18.91		dmn	vis	15.22		dmn	vis	13.74
cen	dan	18.91		sal	dan	15.21		aud	sal	13.74
dmn	cen	18.79		ssh	cen	15.18		dmn	sal	13.74
vis	sal	18.77		ssh	sal	15.17		ssh	dmn	13.73
dmn	sal	18.67		vis	sal	15.16		vis	vis	13.71
mem	cen	18.62		vis	vis	15.14		ssh	dmn	13.70
vis	vis	18.58		vis	van	15.13		mem	van	13.70
dmn	vis	18.55		sal	van	15.13		dmn	vis	13.69
vis	vis	18.51		vis	cen	15.12		vis	sal	13.68
dmn	dmn	18.44		vis	sal	15.12		dmn	sal	13.68
vis	vis	18.44		vis	cen	15.11		vis	sal	13.68
vis	vis	18.40		sal	sub	15.11		unc	vis	13.65
dmn	vis	18.37		dmn	vis	15.10		cen	sal	13.65
vis	cen	18.34		dmn	vis	15.10		vis	cen	13.62
dmn	vis	18.28		vis	vis	15.10		vis	cen	13.61
dmn	mem	18.23		vis	cen	15.07		dmn	dan	13.61
vis	cen	18.18		vis	sal	15.06		cen	sal	13.60
dmn	cen	18.10		dmn	mem	15.04		dmn	cen	13.58
cen	sal	18.08		dmn	vis	15.04		vis	sal	13.58
dmn	vis	18.02		ssh	dmn	15.03		cen	unc	13.58
vis	cen	18.01		vis	vis	15.03		dmn	sal	13.57
cen	sal	17.92		vis	vis	15.01		vis	sal	13.57
cen	sal	17.90		mem	cen	15.01		ssh	vis	13.54
dmn	vis	17.88		dmn	cen	14.99		dmn	cen	13.54
vis	cen	17.88		vis	sal	14.99		vis	cen	13.53
dmn	sal	17.86		dmn	sal	14.99		dmn	sal	13.51
dmn	dmn	17.83		mem	sal	14.99		vis	sal	13.51
vis	vis	17.81		vis	sal	14.99		dmn	vis	13.50
vis	sal	17.77		dmn	cen	14.97		sub	van	13.49
vis	cen	17.74		unc	vis	14.96		dmn	cen	13.47
vis	unc	17.61		vis	vis	14.95		unc	dan	13.47
vis	cen	17.58		vis	vis	14.94		con	con	13.45
dmn	vis	17.54		vis	cen	14.94		mem	sal	13.45
dmn	van	17.52		vis	sal	14.93		mem	van	13.44
unc	dmn	17.51		sal	dan	14.93		dmn	van	13.43
vis	vis	17.51		vis	vis	14.91		vis	vis	13.41
dmn	mem	17.48		dmn	dan	14.90		vis	cen	13.41
dmn	vis	17.42		ssh	sal	14.83		cen	sal	13.41
dmn	cen	17.42		vis	vis	14.81		dmn	dan	13.41
vis	dan	17.42		dmn	cen	14.81		ssh	dan	13.40
vis	dan	17.35		dmn	dan	14.81		vis	dan	13.40
con	dmn	17.30		mem	vis	14.77		vis	sal	13.37

unc	dan	17.30		vis	sal	14.76		ssh	sal	13.36
con	con	17.29		vis	cen	14.74		vis	vis	13.35
ssh	dmn	17.28		vis	vis	14.73		cen	sal	13.35
vis	vis	17.19		vis	van	14.73		dmn	sal	13.32
dmn	cen	17.16		vis	unc	14.72		vis	sal	13.32
ssh	cen	17.14		vis	vis	14.71		dmn	vis	13.31
dmn	cen	17.13		dmn	vis	14.70		unc	ssh	13.30
vis	dan	17.13		vis	sal	14.70		ssh	unc	13.27
dmn	vis	17.10		dmn	vis	14.69		dmn	vis	13.26
vis	sal	17.05		dmn	mem	14.68		ssh	sal	13.26
ssh	dmn	16.99		dmn	vis	14.68		dmn	cen	13.25
sal	sub	16.99		cen	dan	14.65		con	aud	13.24
vis	dan	16.99		vis	unc	14.64		mem	cen	13.24
ssh	dmn	16.98		vis	dan	14.62		vis	dan	13.23
cen	dan	16.97		vis	dan	14.61		dmn	vis	13.22
dmn	dmn	16.96		vis	sal	14.60		con	aud	13.21
vis	cen	16.92		unc	vis	14.59		ssh	dmn	13.21
vis	dan	16.92		dmn	vis	14.59		vis	vis	13.21
vis	vis	16.91		vis	cen	14.58		dmn	dan	13.21
vis	sal	16.90		ssh	dmn	14.57		ssh	sal	13.20
ssh	vis	16.88		ssm	sal	14.57		vis	cen	13.19
dmn	dmn	16.87		dmn	vis	14.56		vis	sal	13.18
vis	vis	16.82		mem	vis	14.56		mem	cen	13.17
dmn	cen	16.77		dmn	dan	14.56		dmn	vis	13.16
vis	cen	16.77		vis	vis	14.55		dmn	cen	13.16
mem	vis	16.72		unc	dmn	14.54		ssh	sal	13.16
cen	sal	16.72		dmn	vis	14.52		dmn	vis	13.15
dmn	sal	16.72		vis	cen	14.50		ssh	vis	13.14
dmn	dmn	16.65		dmn	cen	14.49		vis	cen	13.14
vis	vis	16.62		vis	sal	14.46		dmn	dan	13.14
con	dmn	16.60		dmn	dmn	14.45		mem	vis	13.12
sal	van	16.58		dmn	sal	14.45		vis	cen	13.12
mem	unc	16.54		dmn	sal	14.44		dmn	sal	13.12
mem	sal	16.50		dmn	vis	14.43		vis	dan	13.11
vis	dan	16.50		dmn	vis	14.43		dmn	dan	13.10
vis	vis	16.49		dmn	van	14.43		dmn	vis	13.09
cen	cen	16.46		dmn	vis	14.42		aud	vis	13.09
dmn	sal	16.44		sal	van	14.42		dmn	dan	13.09
dmn	sal	16.43		sal	dan	14.40		dmn	vis	13.08
dmn	cen	16.41		vis	cen	14.39		ssh	sal	13.08
vis	cen	16.40		vis	cen	14.39		dmn	cen	13.07
cen	sal	16.33		vis	dan	14.37		mem	cen	13.07
vis	cen	16.29		ssm	con	14.35		mem	cen	13.07
cen	cen	16.25		dmn	mem	14.35		dmn	sal	13.07
dmn	dan	16.25		dmn	vis	14.35		ssm	dmn	13.06
vis	sal	16.23		van	dan	14.34		dmn	dmn	13.06
sal	dan	16.20		cen	dan	14.34		con	sal	13.06
vis	vis	16.17		dmn	vis	14.33		dmn	vis	13.04
vis	sal	16.17		dmn	cen	14.32		vis	sal	13.04
dmn	dan	16.15		vis	sal	14.31		con	dan	13.04

dmn	vis	16.11		dan	dan	14.31		vis	dan	13.04
vis	vis	16.09		dmn	dmn	14.28		aud	vis	13.03
con	van	16.08		vis	vis	14.27		dmn	cen	13.03
dmn	sal	16.07		con	aud	14.25		dmn	vis	13.01
ssm	dmn	16.05		dmn	vis	14.25		sal	sal	13.01
dmn	vis	16.03		vis	van	14.25				

[a]NBS F-test at T = 13.0; $P < 0.05$ with 10,000 permutations, adjusted for multiple comparisons. [b]Stats indicates the T-statistic of significant edges. Abbreviations; AUD auditory network; CEN central-executive network; CER cerebellum; CON cingulo-opercular network; DAN dorsal attention network; DMN default mode network; MEM memory; RSN Resting State Network; SAL salience; SSH somatosensory hand; SSM somatosensory mouth; SUB subcortical; VAN ventral attention network; VIS visual.

Supplementary Table 12. Results of the network based statistics for bipolar-I disorder patients with hallucinations versus healthy controls using the Power atlas[a].

Bipolar-I disorder patients with hallucinations versus healthy controls										
**RSN**	**RSN**	**Stats**		**RSN**	**RSN**	**Stats**		**RSN**	**RSN**	**Stats**
vis	unc	24.89		vis	unc	12.25		dmn	dan	10.39
vis	unc	23.13		vis	unc	12.23		mem	unc	10.36
vis	unc	22.90		vis	vis	12.19		ssh	dmn	10.34
vis	unc	22.18		unc	cen	12.19		con	aud	10.32
vis	unc	21.96		mem	vis	12.07		unc	dmn	10.31
vis	unc	21.92		vis	unc	12.04		unc	dan	10.31
vis	unc	21.80		mem	dan	12.04		con	sub	10.28
vis	unc	21.01		mem	unc	11.99		vis	unc	10.27
ssm	ssm	20.21		vis	vis	11.83		con	sal	10.24
vis	unc	19.42		sub	sub	11.82		dmn	unc	10.20
vis	unc	19.38		dmn	sal	11.73		dmn	vis	10.18
vis	unc	19.29		aud	dmn	11.71		vis	vis	10.18
dmn	unc	19.15		dmn	dan	11.68		dmn	dmn	10.16
vis	vis	19.03		cen	sub	11.64		unc	van	10.14
vis	unc	18.85		ssh	aud	11.63		dmn	unc	10.13
dmn	vis	18.49		con	dmn	11.62		sal	sal	10.12
vis	unc	17.91		unc	dan	11.62		vis	unc	10.09
dmn	vis	17.80		unc	vis	11.61		con	sub	10.04
vis	unc	17.52		unc	vis	11.56		vis	vis	10.00
dmn	vis	17.29		dmn	vis	11.55		mem	cen	9.96
vis	vis	17.14		ssh	unc	11.52		vis	vis	9.94
vis	unc	16.93		mem	vis	11.51		dmn	vis	9.93
vis	unc	16.33		dmn	vis	11.49		ssm	aud	9.89
ssm	aud	16.28		dmn	vis	11.48		vis	vis	9.87
vis	vis	16.25		ssm	aud	11.44		aud	cen	9.86
mem	dan	16.22		dmn	cen	11.41		dmn	unc	9.82
dmn	vis	16.15		dmn	cen	11.38		aud	dmn	9.81
ssm	aud	16.00		vis	unc	11.37		ssm	aud	9.80
con	unc	15.95		dmn	vis	11.36		cen	sal	9.77
vis	vis	15.94		dmn	vis	11.36		aud	vis	9.76
mem	dan	15.90		vis	unc	11.32		dmn	vis	9.76
dmn	vis	15.84		dmn	dmn	11.31		dmn	vis	9.75
dmn	vis	15.79		dmn	unc	11.30		mem	vis	9.75
dmn	unc	15.76		aud	cen	11.28		ssh	mem	9.70
vis	unc	15.75		dmn	unc	11.27		mem	mem	9.68
vis	unc	15.60		dmn	dmn	11.26		dmn	vis	9.67
vis	unc	15.56		aud	dan	11.25		vis	unc	9.66
mem	vis	15.53		mem	vis	11.24		mem	vis	9.64
vis	unc	15.48		dmn	vis	11.22		vis	vis	9.63
dmn	vis	15.39		dmn	unc	11.20		aud	cen	9.63
mem	vis	15.35		unc	unc	11.20		dmn	unc	9.63
vis	unc	15.29		ssm	aud	11.19		sal	sal	9.62
dmn	unc	15.03		vis	vis	11.19		vis	vis	9.61
dmn	unc	14.89		dmn	unc	11.19		vis	vis	9.59
mem	vis	14.83		vis	unc	11.18		mem	mem	9.57
dmn	vis	14.72		dmn	dmn	11.16		dmn	dmn	9.56
ssh	aud	14.63		mem	vis	11.14		unc	vis	9.56

dmn	vis	14.61		mem	vis	11.13		ssh	unc	9.56
vis	unc	14.60		vis	unc	11.13		vis	vis	9.49
mem	unc	14.34		vis	cen	11.13		ssh	unc	9.49
ssm	aud	14.25		vis	vis	11.11		vis	vis	9.47
ssh	unc	14.17		ssh	ssm	11.10		dmn	vis	9.44
sal	sal	13.86		aud	cen	11.04		unc	dan	9.43
vis	vis	13.81		dmn	dmn	11.03		mem	cen	9.42
dmn	unc	13.74		vis	unc	11.03		vis	vis	9.40
dmn	dmn	13.70		mem	dan	10.99		dmn	dan	9.40
con	cen	13.68		vis	vis	10.97		aud	dmn	9.39
vis	vis	13.66		vis	unc	10.97		unc	vis	9.33
vis	vis	13.66		ssh	aud	10.96		ssh	dmn	9.32
vis	vis	13.65		vis	unc	10.95		dmn	unc	9.32
mem	vis	13.38		unc	vis	10.93		mem	unc	9.32
dmn	unc	13.37		unc	dan	10.92		dmn	dmn	9.31
cen	sal	13.25		mem	vis	10.89		unc	cen	9.31
dmn	vis	13.24		dmn	vis	10.88		aud	dmn	9.30
unc	unc	13.24		dmn	mem	10.87		vis	vis	9.29
dmn	vis	13.06		ssm	aud	10.85		aud	cen	9.29
vis	vis	12.99		vis	vis	10.85		dmn	dmn	9.25
dmn	cen	12.94		vis	unc	10.84		vis	vis	9.22
mem	cen	12.93		mem	vis	10.83		dmn	cen	9.22
mem	dan	12.92		dmn	dmn	10.82		vis	van	9.21
mem	sal	12.87		vis	dan	10.80		unc	vis	9.20
dmn	mem	12.80		vis	vis	10.79		dmn	vis	9.19
cen	sal	12.79		aud	unc	10.79		dmn	dmn	9.17
dmn	dmn	12.75		ssm	ssm	10.78		unc	van	9.16
vis	unc	12.70		dmn	vis	10.75		vis	unc	9.14
vis	cen	12.68		vis	vis	10.74		ssh	unc	9.14
vis	vis	12.64		vis	vis	10.73		con	dmn	9.12
vis	cen	12.58		dmn	vis	10.66		vis	dan	9.12
sal	sal	12.58		ssh	cen	10.58		vis	vis	9.10
sal	sal	12.56		vis	vis	10.55		vis	vis	9.08
dmn	vis	12.48		vis	vis	10.55		unc	mem	9.08
dmn	mem	12.46		unc	unc	10.49		mem	unc	9.06
dmn	vis	12.44		dmn	unc	10.47		dmn	sal	9.04
dmn	cen	12.44		con	sal	10.47		dmn	mem	9.03
dmn	vis	12.43		con	vis	10.44		ssh	ssm	9.01
vis	unc	12.30		vis	vis	10.43		dmn	vis	9.01
aud	dmn	12.29		dmn	vis	10.42		dmn	vis	9.01
ssm	aud	12.25								

[a]NBS F-test at T = 12.0; $P < 0.05$ with 10,000 permutations, adjusted for multiple comparisons. [b]Stats indicates the T-statistic of significant edges. Abbreviations; AUD auditory network; CEN central-executive network; CER cerebellum; CON cingulo-opercular network; DAN dorsal attention network; DMN default mode network; MEM memory; RSN Resting State Network; SAL salience; SSH somatosensory hand; SSM somatosensory mouth; SUB subcortical; VAN ventral attention network; VIS visual.

Supplementary Table 13. List of features for random forest classifier in non-clinical individuals with hallucinations[a].

Non-clinical individuals with hallucinations versus healthy controls					
**No.**	**Region**	**Region**	**No.**	**Region**	**Region**
1	Fusiform_R	Rolandic_Oper_R	47	Cuneus_R	Frontal_Sup_R
2	Hippocampus_L	Frontal_Inf_Oper_R	48	Cuneus_R	Frontal_Mid_R
3	Hippocampus_L	Rolandic_Oper_R	49	Cuneus_R	Frontal_Sup_Medial_R
4	Temporal_Inf_L	SupraMarginal_R	50	Cuneus_R	Cuneus_L
5	Temporal_Inf_L	Angular_R	51	Lingual_R	Frontal_Sup_R
6	Temporal_Inf_L	Temporal_Sup_R	52	Lingual_R	Frontal_Mid_R
7	Temporal_Inf_R	Rolandic_Oper_R	53	Lingual_R	Frontal_Sup_Medial_L
8	Temporal_Inf_R	Amygdala_R	54	Lingual_R	Frontal_Sup_Medial_R
9	Temporal_Inf_R	Temporal_Sup_R	55	Lingual_R	6_Cingulum_Ant_R
10	Temporal_Mid_L	Rolandic_Oper_R	56	Lingual_R	Calcarine_L
11	Temporal_Mid_L	Temporal_Sup_R	57	Lingual_R	Cuneus_L
12	Temporal_Mid_R	Precentral_R	58	Occipital_Inf_L	Frontal_Sup_Medial_R
13	Temporal_Mid_R	Rolandic_Oper_R	59	Occipital_Inf_L	6_Cingulum_Ant_R
14	Temporal_Mid_R	6_Insula_R	60	Occipital_Mid_L	Frontal_Sup_Medial_R
15	Temporal_Mid_R	Heschl_R	61	Occipital_Mid_L	6_Cingulum_Ant_R
16	Temporal_Mid_R	Temporal_Sup_R	62	Occipital_Mid_L	Calcarine_L
17	Temporal_Pole_Mid_R	Frontal_Sup_L	63	Occipital_Sup_L	Frontal_Sup_Medial_R
18	Temporal_Pole_Sup_R	Temporal_Sup_L	64	Putamen_R	Precentral_L
19	Temporal_Sup_L	Rolandic_Oper_R	65	Putamen_R	SupraMarginal_R
20	Temporal_Sup_L	6_Insula_L	66	Angular_R	Precentral_R
21	Temporal_Sup_L	6_Insula_R	67	Angular_R	Parietal_Inf_L
22	Temporal_Sup_R	Precentral_L	68	Parietal_Inf_L	Precentral_R
23	Temporal_Sup_R	Frontal_Sup_L	69	Parietal_Inf_L	Frontal_Sup_Medial_R
24	Temporal_Sup_R	Rolandic_Oper_R	70	Postcentral_L	Rolandic_Oper_R
25	Temporal_Sup_R	Parietal_Inf_L	71	Postcentral_L	Calcarine_L
26	Temporal_Sup_R	SupraMarginal_L	72	Postcentral_R	Rolandic_Oper_R
27	Temporal_Sup_R	SupraMarginal_R	73	Precuneus_R	Frontal_Sup_Medial_R
28	Frontal_Inf_Oper_R	Precentral_L	74	Precuneus_R	Precuneus_L
29	Frontal_Inf_Orb_L	Precentral_L	75	SupraMarginal_L	Rolandic_Oper_R
30	Frontal_Inf_Orb_R	Precentral_L	76	SupraMarginal_L	6_Insula_L
31	Frontal_Inf_Tri_L	Precentral_L	77	SupraMarginal_R	Precentral_R
32	Frontal_Inf_Tri_R	Precentral_L	78	SupraMarginal_R	Frontal_Sup_R
33	Frontal_Mid_L	Precentral_L	79	SupraMarginal_R	Frontal_Mid_R
34	Frontal_Mid_R	Precentral_L	80	SupraMarginal_R	Frontal_Inf_Oper_R
35	Frontal_Sup_L	Precentral_L	81	SupraMarginal_R	Frontal_Inf_Tri_R
36	Frontal_Sup_Medial_L	Frontal_Sup_R	82	SupraMarginal_R	Rolandic_Oper_R
37	Frontal_Sup_Medial_R	Frontal_Sup_R	83	SupraMarginal_R	6_Insula_R
38	Calcarine_L	Frontal_Sup_L	84	SupraMarginal_R	Hippocampus_R
39	Calcarine_L	Frontal_Sup_Medial_L	85	6_Cingulum_Ant_L	Frontal_Sup_R
40	Calcarine_L	Frontal_Sup_Medial_R	86	6_Cingulum_Ant_L	Frontal_Sup_Medial_L
41	Calcarine_R	Frontal_Sup_R	87	6_Cingulum_Ant_R	Frontal_Sup_R
42	Calcarine_R	Frontal_Sup_Medial_R	88	6_Cingulum_Ant_R	Frontal_Mid_R
43	Cuneus_L	Frontal_Sup_Medial_L	89	6_Cingulum_Ant_R	Frontal_Sup_Medial_R
44	Cuneus_L	Frontal_Sup_Medial_R	90	6_Insula_L	Precentral_L
45	Cuneus_L	6_Cingulum_Ant_R	91	6_Insula_R	Precentral_L
46	Cuneus_L	Calcarine_R			

[a]Features are disturbed connections that are part of the NBS network when comparing non-clinical individuals with hallucinations to healthy controls.

Supplementary Table 14. List of features for random forest classifier in schizophrenia patients with hallucinations[a].

Schizophrenia patients with hallucinations versus healthy controls					
No.	Region	Region	No.	Region	Region
1	Amygdala_R	Hippocampus_R	45	Occipital_Mid_L	Lingual_L
2	Amygdala_R	ParaHippocampal_R	46	Occipital_Sup_L	Frontal_Sup_Orb_L
3	Fusiform_L	Occipital_Sup_L	47	Occipital_Sup_L	Lingual_L
4	Heschl_L	Rolandic_Oper_R	48	Caudate_R	Cingulum_Mid_R
5	Heschl_L	Insula_L	49	Caudate_R	Hippocampus_R
6	Heschl_L	Amygdala_R	50	Pallidum_R	Hippocampus_R
7	Heschl_R	Parietal_Inf_R	51	Pallidum_R	ParaHippocampal_R
8	Heschl_R	SupraMarginal_L	52	Putamen_L	Hippocampus_L
9	Heschl_R	SupraMarginal_R	53	Putamen_L	Hippocampus_R
10	Hippocampus_R	Frontal_Inf_Oper_R	54	Putamen_R	Rolandic_Oper_L
11	Hippocampus_R	Supp_Motor_Area_R	55	Putamen_R	Hippocampus_R
12	Hippocampus_R	Cingulum_Ant_L	56	Thalamus_R	Frontal_Inf_Tri_L
13	Hippocampus_R	Cingulum_Ant_R	57	Thalamus_R	Cingulum_Ant_R
14	Hippocampus_R	Cingulum_Mid_R	58	Parietal_Inf_L	Frontal_Med_Orb_L
15	ParaHippocampal_R	Frontal_Inf_Oper_R	59	Parietal_Inf_L	Frontal_Med_Orb_R
16	ParaHippocampal_R	Cingulum_Ant_L	60	Parietal_Inf_L	Lingual_L
17	ParaHippocampal_R	Cingulum_Ant_R	61	Postcentral_L	Supp_Motor_Area_L
18	Temporal_Inf_L	Rolandic_Oper_R	62	Postcentral_L	Supp_Motor_Area_R
19	Temporal_Inf_L	Heschl_R	63	Precuneus_L	Frontal_Sup_Orb_L
20	Temporal_Inf_L	Temporal_Pole_Sup_R	64	Precuneus_R	Frontal_Sup_Orb_L
21	Temporal_Inf_R	Rolandic_Oper_R	65	Precuneus_R	Insula_R
22	Temporal_Inf_R	Heschl_R	66	SupraMarginal_L	Cingulum_Mid_R
23	Temporal_Mid_L	Rolandic_Oper_R	67	SupraMarginal_R	Frontal_Inf_Oper_R
24	Temporal_Mid_L	Lingual_L	68	SupraMarginal_R	Frontal_Inf_Tri_L
25	Temporal_Mid_R	Rolandic_Oper_R	69	SupraMarginal_R	Rolandic_Oper_R
26	Temporal_Sup_L	Rolandic_Oper_R	70	SupraMarginal_R	Insula_R
27	Temporal_Sup_L	Supp_Motor_Area_L	71	SupraMarginal_R	Cingulum_Mid_R
28	Temporal_Sup_L	Supp_Motor_Area_R	72	Cingulum_Mid_R	Precentral_L
29	Temporal_Sup_L	Insula_L	73	Cingulum_Mid_R	Frontal_Sup_Orb_L
30	Temporal_Sup_L	Amygdala_R	74	Cingulum_Mid_R	Frontal_Sup_Orb_R
31	Frontal_Sup_Orb_L	Frontal_Sup_L	75	Cingulum_Mid_R	Frontal_Mid_L
32	Rolandic_Oper_L	Frontal_Inf_Tri_L	76	Cingulum_Mid_R	Frontal_Mid_R
33	Rolandic_Oper_L	Frontal_Inf_Orb_L	77	Cingulum_Mid_R	Frontal_Mid_Orb_R
34	Rolandic_Oper_L	Frontal_Inf_Orb_R	78	Cingulum_Mid_R	Frontal_Inf_Oper_L
35	Rolandic_Oper_R	Rolandic_Oper_L	79	Cingulum_Mid_R	Frontal_Inf_Oper_R
36	Cuneus_L	Frontal_Sup_Orb_L	80	Cingulum_Mid_R	Frontal_Inf_Tri_L
37	Cuneus_R	Frontal_Sup_L	81	Cingulum_Mid_R	Frontal_Inf_Orb_R
38	Cuneus_R	Frontal_Sup_Orb_L	82	Cingulum_Mid_R	Rolandic_Oper_L
39	Cuneus_R	Rectus_L	83	Cingulum_Mid_R	Rolandic_Oper_R
40	Lingual_L	Frontal_Sup_Orb_L	84	Cingulum_Mid_R	Cingulum_Ant_R
41	Occipital_Mid_L	Frontal_Sup_R	85	Cingulum_Post_L	Frontal_Inf_Oper_R
42	Occipital_Mid_L	Frontal_Sup_Orb_L	86	Insula_L	Rolandic_Oper_L
43	Occipital_Mid_L	Frontal_Sup_Medial_L	87	Insula_R	Rolandic_Oper_L
44	Occipital_Mid_L	Rectus_L			

[a]Features are disturbed connections that are part of the NBS network when comparing schizophrenia patients with hallucinations to healthy controls.

Supplementary Table 15. List of features for random forest classifier in bipolar-I disorder patients with hallucinations[a].

Bipolar-I disorder patients with hallucinations versus healthy controls					
**No**	**Region**	**Region**	**No**	**Region**	**Region**
1	Amygdala_L	Frontal_Sup_R	50	Rolandic_Oper	Frontal_Inf_Oper_L
2	Amygdala_L	Frontal_Sup_Orb_L	51	Rolandic_Oper	Frontal_Inf_Oper_R
3	Amygdala_L	Frontal_Sup_Orb	52	Occipital_Inf_R	Frontal_Sup_Orb_R
4	Amygdala_L	Frontal_Mid_L	53	Occipital_Inf_R	Cingulum_Post_L
5	Amygdala_L	Frontal_Mid_R	54	Occipital_Inf_R	Hippocampus_R
6	Amygdala_L	Frontal_Mid_Orb_R	55	Occipital_Mid_R	Cingulum_Post_L
7	Amygdala_L	Frontal_Inf_Oper	56	Occipital_Mid_R	Occipital_Sup_R
8	Amygdala_L	Frontal_Inf_Tri_R	57	Caudate_L	Frontal_Mid_L
9	Amygdala_L	Frontal_Inf_Orb_L	58	Caudate_L	Frontal_Mid_Orb_L
10	Amygdala_L	Frontal_Inf_Orb_R	59	Caudate_R	Frontal_Mid_L
11	Amygdala_L	Rolandic_Oper_R	60	Caudate_R	Frontal_Mid_Orb_L
12	Amygdala_L	Insula_R	61	Caudate_R	Frontal_Inf_Orb_L
13	Amygdala_L	Cingulum_Ant_R	62	Caudate_R	Frontal_Sup_Medial
14	Fusiform_R	Calcarine_R	63	Caudate_R	Cingulum_Ant_L
15	Heschl_R	Precentral_R	64	Caudate_R	Cingulum_Ant_R
16	Heschl_R	Frontal_Inf_Oper_L	65	Caudate_R	Fusiform_R
17	Heschl_R	Frontal_Inf_Tri_L	66	Caudate_R	Parietal_Inf_R
18	Hippocampus_R	Rolandic_Oper_R	67	Caudate_R	Angular_R
19	Temporal_Inf_L	Rolandic_Oper_R	68	Pallidum_L	Frontal_Mid_L
20	Temporal_Inf_L	Caudate_R	69	Pallidum_L	Frontal_Mid_Orb_L
21	Temporal_Mid_R	Caudate_R	70	Pallidum_L	Frontal_Mid_Orb_R
22	Temporal_Pole_Mid	Putamen_L	71	Pallidum_L	Frontal_Inf_Oper_L
23	Temporal_Pole_Mid	Pallidum_L	72	Pallidum_L	Frontal_Inf_Oper_R
24	Temporal_Pole_Sup	Frontal_Mid_L	73	Pallidum_L	Rolandic_Oper_R
25	Temporal_Pole_Sup	Frontal_Mid_Orb_L	74	Pallidum_L	Olfactory_R
26	Temporal_Pole_Sup	Rolandic_Oper_R	75	Pallidum_R	Frontal_Mid_L
27	Temporal_Pole_Sup	Pallidum_L	76	Pallidum_R	Parietal_Inf_R
28	Temporal_Sup_L	Precentral_R	77	Putamen_L	Frontal_Mid_L
29	Frontal_Inf_Oper_R	Frontal_Mid_Orb_L	78	Putamen_L	Frontal_Mid_Orb_L
30	Frontal_Inf_Orb_L	Frontal_Sup_L	79	Putamen_L	Frontal_Inf_Oper_L
31	Frontal_Inf_Orb_L	Frontal_Mid_L	80	Putamen_L	Frontal_Inf_Oper_R
32	Frontal_Inf_Orb_L	Frontal_Mid_R	81	Putamen_L	Frontal_Inf_Tri_L
33	Frontal_Inf_Orb_L	Frontal_Mid_Orb_L	82	Putamen_L	Rolandic_Oper_R
34	Frontal_Inf_Tri_L	Frontal_Mid_Orb_L	83	Putamen_L	Olfactory_R
35	Frontal_Med_Orb_L	Frontal_Mid_Orb_L	84	Putamen_L	Caudate_R
36	Frontal_Med_Orb_R	Frontal_Mid_L	85	Parietal_Inf_R	Frontal_Sup_Medial
37	Frontal_Med_Orb_R	Frontal_Mid_Orb_L	86	Postcentral_L	Precentral_R
38	Frontal_Med_Orb_R	Frontal_Med_Orb	87	Postcentral_R	Precentral_R
39	Frontal_Mid_Orb_L	Frontal_Sup_L	88	Postcentral_R	Postcentral_L
40	Frontal_Mid_Orb_L	Frontal_Sup_R	89	SupraMarginal	Precentral_L
41	Frontal_Mid_Orb_L	Frontal_Mid_L	90	Cingulum_Ant_L	Frontal_Mid_L
42	Frontal_Mid_Orb_L	Frontal_Mid_R	91	Cingulum_Ant_L	Frontal_Mid_Orb_L
43	Frontal_Sup_Medial	Frontal_Mid_Orb_L	92	Cingulum_Ant_L	Frontal_Inf_Orb_L
44	Frontal_Sup_Medial	Frontal_Inf_Orb_L	93	Cingulum_Ant_R	Frontal_Mid_Orb_L
45	Frontal_Sup_Medial	Frontal_Inf_Orb_L	94	Cingulum_Ant_R	Frontal_Inf_Orb_L
46	Precentral_R	Precentral_L	95	Cingulum_Mid	Frontal_Mid_Orb_L
47	Rolandic_Oper_L	Precentral_R	96	Insula_L	Frontal_Mid_Orb_L
48	Rolandic_Oper_L	Frontal_Mid_L	97	Insula_L	Frontal_Inf_Oper_R
49	Rolandic_Oper_L	Frontal_Mid_R			

[a]Features are disturbed connections that are part of the NBS network when comparing bipolar-I patients with hallucinations to healthy controls.

Supplementary Table 16. List of abbreviations for brain areas AAL-atlas[a].

No.	Abbreviation	Region	No.	Abbreviation	Region
1	PreCG.L	Precentral_L	46	CUN.R	Cuneus_R
2	PreCG.R	Precentral_R	47	LING.L	Lingual_L
3	SFGdor.L	Frontal_Sup_L	48	LING.R	Lingual_R
4	SFGdor.R	Frontal_Sup_R	49	SOG.L	Occipital_Sup_L
5	ORBsup.L	Frontal_Sup_Orb_L	50	SOG.R	Occipital_Sup_R
6	ORBsup.R	Frontal_Sup_Orb_R	51	MOG.L	Occipital_Mid_L
7	MFG.L	Frontal_Mid_L	52	MOG.R	Occipital_Mid_R
8	MFG.R	Frontal_Mid_R	53	IOG.L	Occipital_Inf_L
9	ORBmid.L	Frontal_Mid_Orb_L	54	IOG.R	Occipital_Inf_R
10	ORBmid.R	Frontal_Mid_Orb_R	55	FFG.L	Fusiform_L
11	IFGoperc.L	Frontal_Inf_Oper_L	56	FFG.R	Fusiform_R
12	IFGoperc.R	Frontal_Inf_Oper_R	57	PoCG.L	Postcentral_L
13	IFGtriang.L	Frontal_Inf_Tri_L	58	PoCG.R	Postcentral_R
14	IFGtriang.R	Frontal_Inf_Tri_R	59	SPG.L	Parietal_Sup_L
15	ORBinf.L	Frontal_Inf_Orb_L	60	SPG.R	Parietal_Sup_R
16	ORBinf.R	Frontal_Inf_Orb_R	61	IPL.L	Parietal_Inf_L
17	ROL.L	Rolandic_Oper_L	62	IPL.R	Parietal_Inf_R
18	ROL.R	Rolandic_Oper_R	63	SMG.L	SupraMarginal_L
19	SMA.L	Supp_Motor_Area_L	64	SMG.R	SupraMarginal_R
20	SMA.R	Supp_Motor_Area_R	65	ANG.L	Angular_L
21	OLF.L	Olfactory_L	66	ANG.R	Angular_R
22	OLF.R	Olfactory_R	67	PCUN.L	Precuneus_L
23	SFGmed.L	Frontal_Sup_Medial_L	68	PCUN.R	Precuneus_R
24	SFGmed.R	Frontal_Sup_Medial_R	69	PCL.L	Paracentral_Lobule_L
25	ORBsupmed.L	Frontal_Med_Orb_L	70	PCL.R	Paracentral_Lobule_R
26	ORBsupmed.R	Frontal_Med_Orb_R	71	CAU.L	Caudate_L
27	REC.L	Rectus_L	72	CAU.R	Caudate_R
28	REC.R	Rectus_R	73	PUT.L	Putamen_L
29	INS.L	Insula_L	74	PUT.R	Putamen_R
30	INS.R	Insula_R	75	PAL.L	Pallidum_L
31	ACG.L	Cingulum_Ant_L	76	PAL.R	Pallidum_R
32	ACG.R	Cingulum_Ant_R	77	THA.L	Thalamus_L
33	DCG.L	Cingulum_Mid_L	78	THA.R	Thalamus_R
34	DCG.R	Cingulum_Mid_R	79	HES.L	Heschl_L
35	PCG.L	Cingulum_Post_L	80	HES.R	Heschl_R
36	PCG.R	Cingulum_Post_R	81	STG.L	Temporal_Sup_L
37	HIP.L	Hippocampus_L	82	STG.R	Temporal_Sup_R
38	HIP.R	Hippocampus_R	83	TPOsup.L	Temporal_Pole_Sup_L
39	PHG.L	ParaHippocampal_L	84	TPOsup.R	Temporal_Pole_Sup_R
40	PHG.R	ParaHippocampal_R	85	MTG.L	Temporal_Mid_L
41	AMYG.L	Amygdala_L	86	MTG.R	Temporal_Mid_R
42	AMYG.R	Amygdala_R	87	TPOmid.L	Temporal_Pole_Mid_L
43	CAL.L	Calcarine_L	88	TPOmid.R	Temporal_Pole_Mid_R
44	CAL.R	Calcarine_R	89	ITG.L	Temporal_Inf_L
45	CUN.L	Cuneus_L	90	ITG.R	Temporal_Inf_R

[a] Tzourio-Mazoyer and colleagues [58].
